# Comparative clinical-related outcomes of Chinese patent medicines for cardiac hypertrophy: A systematic review and network meta-analysis of randomized clinical trials

**DOI:** 10.3389/fphar.2023.963099

**Published:** 2023-01-23

**Authors:** Tianqi Zhang, Haoyang Xu, Dong Zhen, Danni Fu, Ming Zhao, Chengxi Wei, Xue Bai

**Affiliations:** ^1^ Medical College of Inner Mongolia Minzu University, Tongliao, China; ^2^ Inner Mongolia Key Laboratory of Mongolian Medicine Pharmacology for Cardio-Cerebral Vascular System, Inner Mongolia Minzu University, Tongliao, China; ^3^ Affiliated Hospital of Inner Mongolia Minzu University, Tongliao, China

**Keywords:** cardiac hypertrophy, Qi-replenishing, blood-activating and stasis-removing, Chinese patent medicines, network meta-analysis

## Abstract

**Background:** Persistent pathological cardiac hypertrophy has been associated with increased risk of heart failure and even sudden death. Multiple Chinese patent medicines (CPMs) have gained attention as alternative and complementary remedies due to their high efficiency and few side effects. However, the effects of CPM-related treatment regimens for cardiac hypertrophy had not been systematically evaluated.

**Aim:** The objective of this study was to estimate and compare the effectiveness of different mechanisms of CPMs to improve clinical outcomes, including clinical efficacy and echocardiographic indices, in the treatment of cardiac hypertrophy patents.

**Methods:** A network meta-analysis was conducted on CPM-related randomized controlled trials (RCTs) published between 2012 and 2022 involving cardiac hypertrophy patients from four foreign and four Chinese databases. The outcomes concerned efficacy and related indicators, including echocardiographic indices, cardiac biomarkers, and functional exercise capacity, which were evaluated as odds ratios, mean differences, and 95% credible intervals. Network plots, league tables, surface-under-the-cumulative ranking (SUCRA), and funnel plots were created for each outcome, and all analyses were conducted using Stata 16.0 software.

**Results:** A total of 25 RCTs were evaluated; these involved 2395 patients in a network meta-analysis (NMA). The results from existing evidence indicate that blood-activating and stasis-removing Chinese patent medicine (BASR-CPM) + Western medicine (WM) showed a good improvement in clinical efficacy (OR = 8.27; 95%CI = 0.97, 70.73). A combined treatment regimen of CPM with a function of *qi*-replenishing, blood-activating and stasis-removing, and Western medicine was an effective treatment regimen for echocardiographic indices such as decreasing left ventricular end-systolic dimension (LVESD) (SMD = −2.35; 95%CI = −3.09, −1.62) and left ventricular mass index (LVMI) (SMD = −1.73; 95%CI = −2.92, −0.54). Furthermore, KWYR-CPM + WM and BASR-CPM also showed good improvement for echocardiographic indices of LVEDD (SMD = −1.84; 95%CI = −3.46, −0.22) and left ventricular ejection fraction (SMD = 1.90; 95%CI = −0.46, −3.35), respectively.

**Conclusion:** The study showed that BASR-CPM + WM may be the potentially superior treatment regimen for improving clinical efficacy among cardiac hypertrophy patients. QR&BASR-CPM + WM might be the optimal treatment for decreasing LVESD and LVMI. However, due to potential risks from bias and limited RCTs, further studies with larger samples and high-quality RCTs are needed to support these findings.

**Systematic Review Registration:** [https://www.crd.york.ac.uk/prospero/display_record.php?RecordID=329589],identifier [CRD42022329589].

## Introduction

Heart failure (HF) is a complex disease that seriously threatens human health, mainly manifesting in cardiac structural or functional dysfunction, and impaired ventricular filling or blood ejection (Beldhuis et al., 2022). In recent years, there have been more than 40 million HF patients worldwide, nearly half of whom died within 5 years of diagnosis. To some extent, the mortality rate of HF exceeds that of many cancers (Murphy et al., 2020).

HF is closely related to cardiac hypertrophy, which is the initial adaptive response of the heart to maintain cardiac function under physiological and pathological overload. Cardiac hypertrophy is characterized by an increased volume and mass of cardiomyocytes: the total amount of cardiomyocytes increases and their contractility is enhanced so that the heart can maintain normal contractility. This comprehensive and in-depth investigation of the mechanism of cardiac hypertrophy will contribute to preventing and controlling the occurrence and development of cardiac hypertrophy in its early stage and will have important practical significance for the prevention and treatment of heart failure. Cardiac hypertrophy is divided into physiological and pathological cardiac hypertrophy; persistent pathological myocardial hypertrophy is associated with an increased risk of heart failure and even sudden death.

At present, Western medicine (WM) and traditional Chinese medicine (TCM) are widely applied in inhibiting cardiac hypertrophy (Yokota et al., 2014). According to treatment guidelines, the main drugs in WM for the clinical treatment of HF are angiotensin receptor blockers (ARBs), angiotensin-converting enzyme inhibitors (ACEIs), statins, and mineralocorticoid receptor antagonists (MRAs) (Kuno et al., 2020; Chen et al., 2022). Previous studies have shown that ACEI/ARBs can inhibit cardiac hypertrophy by inhibiting the activity of matrix metalloproteinase (MMP) in plasma, while statin therapy can reduce cardiac hypertrophy by recovering the coronary endothelial function through endogenous nitric oxide for improving long-term clinical efficiency and related clinical outcomes (Yokota et al., 2010; Ishida et al., 2012). However, some studies have shown that a large dose of ARBs could cause AngII accumulation to directly activate AT2R in the body, which could increase the risk of cardiovascular events (Solomon et al., 2011). In addition, ACEIs can induce an irritating dry cough and even nausea and vomiting among patients (Ren et al., 2010). At present, there is increasing *in vitro* and *in vivo* research into the treatment of cardiac hypertrophy with Chinese patent medicine (CPM) (Zhang et al., 2021). Studies have shown that CPM can effectively target cardiac hypertrophy by the regulatory mechanism of TGF-β1 and CTGF to relieve the cardiac fibrosis process (Li et al., 2021; Lv et al., 2021). Hence, the evidence for the combination therapy of TCM and WM indicates that it not only directly acts on the lesion but balances the whole body to improve clinical efficacy and achieve simultaneous treatment at the root. Combined TCM and WM could improve clinical efficacy by as much as 125% compared to just WM in aspects of echocardiographic indices (Zhang et al., 2021). This further indicates that the effectiveness of TCM-related combined treatment can have better long-term and multiple superior effects than conventional WM, especially for improving LVEF and E/A, and reducing SV and serum levels of BNP and CRP (Zhang et al., 2021). However, there is no systematic evaluation of CPM with different mechanisms to treat different clinical outcomes among patients with cardiac hypertrophy, which significantly limits their reliability and popularization in clinical practice (Zhang et al., 2021).

Meta-analysis could get close to real statistical analyses from random controlled trials which have been widely used (Mbuagbaw and Aves, 2022). Conventional meta-analysis on the treatment effects of drugs is conducted on the effect size based on pairwise head-to-head direct comparison but is limited by fewer direct comparisons. Therefore, the need for both direct and indirect comparisons of various drugs of the same efficacy used in clinical practice has received increased attention. Accordingly, network meta-analysis (NMA) is an approach that could directly and indirectly compare any comparative evidence based on logical inference (Shim et al., 2017). Therefore, with the development of research, traditional meta-analysis is being replaced by NMA (Watt and Del Giovane, 2022). In order to more accurately estimate the effects of CPM, we explore the consistency of research evidence and the differing efficacy of all the outcome indicators between CPM which has been limited in the previous literature. In our study, a systematic review and NMA of randomized clinical trials was made to compare clinical outcomes, including efficacy and echocardiographic indices, of CPM related to cardiac hypertrophy. Based on this, better designed trials and more detailed clinical outcomes like safety and efficacy are required to further validate their potential effectiveness from the point of TCM in clinical application to treat cardiomyopathies. Our findings should provide targeted and valuable references for clinical settings.

## Methods

### Protocol and study registration

This NMA study was performed following the Preferred Reporting Items for Systematic Reviews and Meta-Analysis (PRISMA) guidelines Supplementary Material Appendix 1 [Registration ID: CRD42022329589].

### Literature search

The literature search was performed using electronic network databases, including PubMed, EMBASE, Web of Science, the Cochrane Library, China National Knowledge Infrastructure (CNKI), China Biology Medicine disc (CBM), the Information Resource Integration Service Platform (VIP), and the Wanfang Data Knowledge Service Platform (Wanfang Data). All the researched articles were published from 2012 to 2022. The retrieval terms were MeSH subject words and free words such as “cardiac hypertrophy,” “Chinese patent medicine,” and “Randomized Controlled Trials (RCTs)” (for full details, see Supplementary Appendix S2).

### Inclusion and exclusion criteria

The inclusion criteria used for selection studies were

1) patients included in the study were diagnosed with cardiomyopathies, including hypertrophic, familial or hypertrophy, left ventricular hypertrophy, cardiomegaly, or cardiomyopathy. The age of selected patients was over 18 years old;
2) treatment regimens in the intervention group (IG) were assigned with either a combination of CPM with WM, or TCM, CPM and WM, or CPM alone;
3) control group (CG) was treated only with WM;
4) all studies with clinical efficacy as the primary outcome indicator were included;
5) all collected studies were limited to RCTs.

The exclusion criteria were

1) research that includes non-RCTs or duplicated papers;
2) research such as systematic reviews, commentaries, case reports, or animal tests;
3) trials with inconsistent study samples or inappropriate study designs;
4) trials which did not provide complete data or information, or where authors failed to reply upon being contacted.

### Outcome indicators

In this study, the primary outcome was clinical efficacy. Secondary outcomes included echocardiographic indices, cardiac biomarkers, and functional exercise capacity. Echocardiographic indices included left ventricular ejection fraction (LVEF), left ventricular end-diastolic dimension (LVEDD), and left ventricular end-systolic dimension (LVESD). Cardiac biomarkers comprised C-reactive protein (CRP) and N-terminal pro-B-type natriuretic peptide (NT-proBNP). Functional exercise capacity was measured by the six-minute walk test (6-MWT), which is a good index for evaluating the exercise endurance for chronic heart failure. In this test, a patient is required to walk as fast as possible in a straight corridor and measure the walking distance for 6 minutes. If they walk less than 150 m, it indicates severe cardiac insufficiency; if they walk 150–450 m, it indicates moderate cardiac insufficiency; if they walk more than 450 m, it is considered mild cardiac insufficiency.

### Data extraction and screening

The selected papers were extracted and imported into Note Express for electronic and manual checks. Two researchers (TQZ and HYX) independently searched, read, and screened the papers according to the aforementioned criteria. Any controversial results were cross-checked and discussed with a third evaluator (MZ) until consistent conclusions and a consensus were reached. The following information was extracted from the final eligible articles and recorded in Microsoft Excel: name of first author, publication year, basic patient characteristic, sample size in each group, type of intervention and control, duration of follow-up time, and before and after treatment outcome data. All outcome parameters were presented as mean ± standard deviation (SD) and median ± quartile range based on the data provided.

### Quality assessment of extract studies

Two authors (TQZ and HYX) independently evaluated researcher bias using Cochrane Collaboration bias risk tools, which included random sequence generation, allocation concealment, double blinding, triple blinding, incomplete data, and selective reporting. Each of these evaluation domains were then categorized as three levels: high, low, or unclear.

### Data analysis

The categorical variables were expressed by the odds ratio (OR) between the groups before and after treatment. The continuity variables were expressed by the standardized mean deviation (SMD) and 95% confidence interval (CI). Network maps were first constructed and analyzed for direct and indirect comparison of each treatment outcome. Next, we performed a standard pairwise meta-analysis as a direct comparison by forest map and league table (for forest maps, see Supplementary Figure S2) to illustrate the differences between each treatment regimen. Finally, the hierarchy of treatment probability was estimated according to the value of surface under the cumulative ranking curves (SUCRA) in which a larger value was regarded as more probably a superior treatment regimen. The closer to 100 in SUCRA, the more useful the treatment regimen is. All analyses were conducted in Stata 16.0. All *p*-values were two-tailed with statistical significance specified at 0.05 and CI computed at the 95% level.

### Publication bias

Funnel plots were used to test publication bias in this study. There was publication bias, which was hard to control when positive data were more likely to be published in journals with similar research papers with statistical significance.

## Results

### Study selection

**FIGURE 1:**
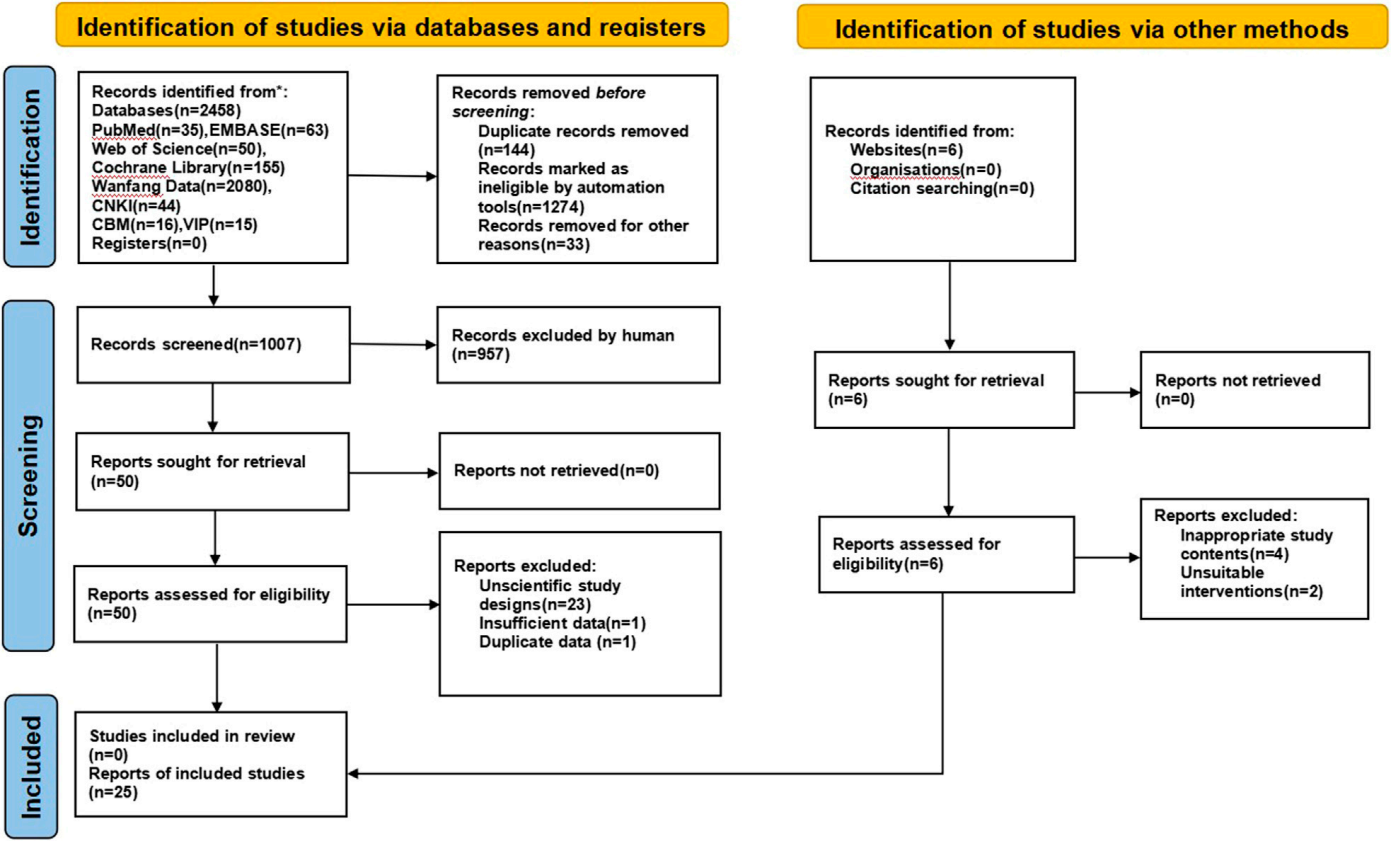
Flowchart of study selection.

The initial search of eight databases yielded 2458 articles, 1451 of which were excluded due to duplicates (*n* = 144), animal tests (*n* = 1274), review articles, and meta-analyses (*n* = 33). After reading the title and abstract, 957 articles were screened out, including inappropriate study contents (*n* = 803) and unsuitable interventions (*n* = 154). Based on our inclusion and exclusion criteria, after reading the full texts manually, 25 articles were excluded for reasons of unscientific study interventions and study design (*n* = 23) or insufficient (*n* = 1) or duplicate data (*n* = 1). Eventually, 25 studies were included in this NMA (Yang et al., 2012; Zheng, 2013; Liu, 2014; Han and Shen, 2015; Cui et al., 2016; Xun, 2016; Zhang et al., 2016; Fan, 2017; Li, 2017; Zhang et al., 2017; Zhou et al., 2018; Wang et al., 2019; Wu and Hu, 2019; Xie et al., 2019; Xu et al., 2019; Zheng et al., 2019; Zou, 2019; Fan et al., 2020; Peng et al., 2020; Yang and Liu, 2020; Hu, 2021; Ji et al., 2021; Meng et al., 2021; Wu et al., 2021; Zhang and Gou, 2021). These were all two-arm studies. The selection process is illustrated in Figure 1.

### Characteristics of included studies

A total of 2,395 patients with cardiac hypertrophy were included in the NMA, with the duration of follow-up ranging from 2 weeks to 1 year. Among them, 1,199 patients were allocated to the intervention group (IG) with CPM-related treatment regimens (261 cases used CPM alone, 902 employed a combination of CPM and WM, and 36 received a combination of CPM, TCM, and WM). A further 1,196 cases were located in the WM-only control group (CG). Detail information for each RCT is shown in Table 1; Table 2 summarizes detailed information about the CPMs in the research articles.

**TABLE 1:** Characteristics of each RCTs in included studies.

No.	Study	Year	Country	Number of participants	Mean age	Male/female		Treatment regimen	Chinese patent medicine of IG and dose	Duration of the follow-up (week)
				(IG/CG)	Mean age of participants	IG	CG	(IG vs. CG)		
					(All or IG/CG)					
1	Zhou et al.	2018	China	66/66	46.06 ± 7.12/45.37 ± 6.81	35/31	38/28	QR-CPM + WM vs. WM	Yiqi Fumai injection, 250–500 ml qd iv	2
2	Wang et al.	2019	China	80/80	48.5 ± 2.4	58/42		QR&BASR-CPM + WM vs. WM	Yixinshu capsule, 3 goals/time tid po	4
3	Xun	2016	China	50/50	49.6 ± 2.3	58/42		QR&BASR-CPM + WM vs. WM	Yixinshu capsule, 3 goals/time tid po	NA
4	Cui et al.	2016	China	50/50	58.1 ± 3.1	55/45		BASR-CPM + WM vs. WM	Xinkeshu capsule, 4 goals/time tid po	24
5	Zheng et al.	2019	China	25/25	57.64 ± 3.97/57.72 ± 4.03	12/13	12/13	QR&BASR-CPM + WM vs. WM	Heart-protecting musk pill, 2 goals/time tid po	12
6	Han and Sen	2015	China	40/40	65.5 ± 2.4/66.0 ± 2.6	22/18	21/19	QR-CPM + WM vs. WM	Qiliqiangxin capsule, 4 goals/time tid po	24
7	Zhang et al.	2016	China	40/40	65.3 ± 2.5/63.0 ± 0.4	21/19	24/16	QR&BASR-CPM + WM vs. WM	Yiqihuayu capsule, 4 goals/time tid po	6
8	Fan et al.	2020	China	64/64	63.8 ± 4.3/63.6 ± 4.5	34/30	33/31	BASR-CPM vs. WM	Ginkgo ester drop pills, 4 goals/time tid po	8
9	Li	2014	China	46/44	68.6 ± 8.4/69.3 ± 3.6	29/17	27/17	QR&BASR-CPM vs. WM	Yiqihuayu capsule, 4 goals/time tid po	6
10	Yang et al.	2012	China	30/29	64.0 ± 11.0/66.0 ± 12.0	17/13	11/18	QR&BASR-CPM + WM vs. WM	Tongxinluo capsule, 3 goals/time tid po	12
11	Wu and Hu	2019	China	86/86	63.9 ± 6.7/65.1 ± 7.5	45/41	49/37	QR&BASR-CPM vs. WM	Musk tongxin dropping pills, 2 goals/time tid po	24
12	Wu et al.	2021	China	65/66	66.52 ± 6.71/66.47 ± 6.53	40/30	43/27	QR&BASR-CPM vs. WM	Musk tongxin dropping pills, 2 goals/time tid po	24
13	Zou	2019	China	71/71	63.24 ± 5.73/63.17 ± 5.49	38/33	37/34	QR&BASR-CPM + WM vs. WM	Heart-protecting musk pill, 2 goals/time tid po	12
14	Yang and Liu	2020	China	43/43	60.14 ± 6.35/61.26 ± 6.27	23/20	19/24	HC-CPM vs. WM	Sanwei sandalwood capsule, 3 goals/time bid po	4
15	Xu et al.	2019	China	40/40	56.85 ± 6.71/55.92 ± 6.64	22/18	24/16	BASR-CPM + WM vs. WM	Compound Danshen dripping pills, 10 goals/time tid po	24
16	Fan	2017	China	40/40	54.2 ± 2.4/53.6 ± 2.7	23/17	25/15	QR-CPM + WM vs. WM	Qiliqiangxin granules, 4 goals/time tid po	12
17	Zhang et al.	2017	China	30/30	64.70 ± 7.82/NA	12/18	14/16	QR-CPM + WM vs. WM	Qiliqiangxin granules, 4 goals/time tid po	4
18	Zheng	2013	China	31/30	73.7 ± 10.6/73.4 ± 9.4	13/18	13/17	QR-CPM + WM vs. WM	Fumai granule, 1 bag/time tid po	4
19	Peng et al.	2020	China	30/30	59.17 ± 9.21/58.22 ± 9.31	17/13	15/15	QR-CPM + WM vs. WM	Jianxin Pinglv pills, 6 g/time tid po	8
20	Hu	2021	China	36/36	61.29 ± 5.22/62.88 ± 5.27	21/15	23/13	KWYR-CPM + THSWT + WM vs. WM	Kidney-qi-tonifying pill, 10 goals/time tid po	12
21	Meng et al.	2021	China	34/34	68.85 ± 4.43/68.48 ± 4.37	17/17	18/16	BASR-CPM + WM vs. WM	Compound Danshen dripping pills, after 20 goals/time tid po, x3D, 10 goals/time tid po	12
22	Ji et al.	2021	China	63/63	75.51 ± 5.22/75.55 ± 5.23	34/29	33/30	QR-CPM + WM vs. WM	Buxinqi oral solution, 10 ml/time tid po	12
23	Liu	2014	China	50/50	50.2 ± 4.1/51.1 ± 4.1	27/23	28/22	QR&BASR-CPM + WM vs. WM	Wenxin granule, 2g/time tid po	4
24	Xie et al.	2019	China	40/40	57.8 ± 8.5/59.6 ± 9.1	10/30	12/28	QR-CPM + WM vs. WM	Qishen Yiqi droplet, 0.5 g/time tid po	48

Notes: ① Clinical efficacy; ② LVEF, left ventricular ejection fraction; ③ LVEDD, left ventricular end-diastolic dimension; ④ LVESD, left ventricular end-systolic dimension; ⑤ LVMI, left ventricular mass index; ⑥ CRP, C-reactive protein; ⑦ NT-proBNP, N-terminal proBNP; ⑧ 6-MWT, six-minute walk test.

**TABLE 2:** Summary of composition of CPMs.

Study	Formulation	Source	Species, concentration	Quality control reported? (Y/N)	Chemical analysis reported? (Y/N)	Therapeutic claims in TCM
Zhou et al. (2018)	Yiqi Fumai injection	[Tianjin Tasly Pride Pharmaceutical Co., Ltd.]	*Talinum paniculatum* (Jacq.) Gaertn. [Talinaceae; Talinum], 0.5g; *Schisandra chinensis* (Turcz.) Baill. [Schisandraceae; Schisandra], and 0.75g; *Ophiopogon japonicus* (Thunb.) Ker Gawl. [Asparagaceae; Ophiopogon], 1.5 g.	Y—Prepared according to the National Food and Drug Administration National Drug Standards	N	Replenishing *qi* and restoring pulse, nourishing *yin*, and engendering liquid
Wang et al. (2019)	Yixinshu capsule	[Guizhou Xinbang Pharmaceutical Co., Ltd.]	*Panax ginseng* C.A.Mey. [Araliaceae; Panax], 200g; *Ophiopogon japonicus* (Thunb.) Ker Gawl. [Asparagaceae; Ophiopogon], 200g; *Schisandra chinensis* (Turcz.) Baill. [Schisandraceae; Schisandra], 133g; *Astragalus mongholicus* Bunge [Fabaceae; Astragalus], 200g; *Salvia miltiorrhiza* Bunge [Lamiaceae; Salvia], 267g; *Conioselinum anthriscoides* ‘Chuanxiong’ [Apiaceae; Conioselinum], 133g; and *Crataegus pinnatifida* Bunge [Rosaceae; Crataegus], 200 g.	Y—Prepared according to People’s Republic of China Pharmacopoeia, 2020	Y—HPLC	Replenishing *qi* and restoring pulse, activating blood, and removing stasis
Xun, (2016)	Yixinshu capsule	[Shandong Zhongtai Pharmaceutical Co. Ltd.]	*Panax ginseng* C.A.Mey. [Araliaceae; Panax], 200g; *Ophiopogon japonicus* (Thunb.) Ker Gawl. [Asparagaceae; Ophiopogon], 200g; *Schisandra chinensis* (Turcz.) Baill. [Schisandraceae; Schisandra], 133g; *Astragalus mongholicus* Bunge [Fabaceae; Astragalus], 200g; *Salvia miltiorrhiza* Bunge [Lamiaceae; Salvia], 267g; *Conioselinum anthriscoides* ‘Chuanxiong’ [Apiaceae; Conioselinum], 133g; and *Crataegus pinnatifida* Bunge [Rosaceae; Crataegus], 200 g.	Y—Prepared according to People’s Republic of China Pharmacopoeia, 2020	Y—HPLC	Replenishing *qi* and restoring pulse, activating blood, and removing stasis
Cui et al. (2016)	Xinkeshu capsule	[Chongqing Xieran Pharmaceutical Co. Ltd.]	*Crataegus pinnatifida* Bunge [Rosaceae; Crataegus], 375g; *Salvia miltiorrhiza* Bunge [Lamiaceae; Salvia], 375g; *Pueraria lobata* (Willd.) Ohwi [Fabaceae; Pueraria], 375g; *Panax notoginseng* (Burkill) F.H.Chen [Araliaceae; Panax], 25g; and *Aucklandia lappa* (Decne.) Decne. [Asteraceae; Dolomiaea], 25 g.	Y—Prepared according to the Ministry of Health of the People’s Republic of China Drug Standards	Y—HPLC	Activating blood and removing stasis, promoting *qi*, and alleviating pain
Zheng et al. (2019)	Heart-protecting musk pill	[Shanghai Hehuang Pharmaceutical Co.Ltd.]	*Moschus* [Cervidae; Moschus]; *Panax ginseng* C.A.Mey. [Araliaceae; Panax]; oriental sweetgum [Hamamelidaceae; *Styralyl propionate*]; bovis calculus [Bovine; *Calculus bovis*]; *Cinnamomum cassia* Siebold [Lauraceae; Cinnamomum]; toad [Bufonidae; toad venom].	Y—Prepared according to People’s Republic of China Pharmacopoeia, 2005	Y—HPLC	Replenishing *qi* and restoring pulse, activating blood, and removing stasis
Han and Shen, (2015)	Qiliqiangxin capsule	[Shijiazhuang Yiling Pharmaceutical Co., Ltd.]	*Astragalus mongholicus* Bunge [Fabaceae; Astragalus], 450g; *Panax ginseng* C.A.Mey. [Araliaceae; Panax], 225g; *Aconitum carmichaelii* Debeaux [Ranunculaceae; Aconitum], 112.5g; *Salvia miltiorrhiza* Bunge [Lamiaceae; Salvia], 225g; *Lepidium apetalum* Willd. [Brassicaceae; Lepidium], 150g; *Alisma plantago-aquatica* L. [Alismataceae; Alisma], 225g; *Polygonatum odoratum* (Mill.) Druce [Asparagaceae; Polygonatum], 75g; *Cinnamomum cassia* Siebold [Lauraceae; Cinnamomum], 90g; *Carthamus tinctorius* L. [Asteraceae; Carthamus], 90g; *Periploca sepium* Bunge [Apocynaceae; Periploca], 180g; and *Citrus reticulata* Blanco [Rutaceae; Citrus], 75 g.	Y—Prepared according to People’s Republic of China Pharmacopoeia, 2020	Y—HPLC	Replenishing *qi* and restoring *yang*, restoring pulse and cardiotonic
Zhang et al. (2016)	Yiqihuayu capsule	[Haisen Pharmaceutical Company of Hebei Medical University, Ltd.]	*Astragalus mongholicus* Bunge [Fabaceae; Astragalus]; *Pseudostellaria* Pax [Caryophyllaceae; Pseudostellaria]; *Conioselinum anthriscoides* ‘Chuanxiong’ [Apiaceae; Conioselinum]; Pheretima [Siliquariidae; Pheretima]; *Paeonia rubra* Steud. [Paeoniaceae; Paeonia]; and *Spatholobus suberectus* Dunn [Fabaceae; Spatholobus].	N	N	Warming kidney and replenishing *qi*, activating blood, and removing stasis
Fan et al. (2020)	Ginkgo ester drop pills	[Zhejiang Jiuxu Pharmaceutical Co. Ltd.]	*Ginkgo biloba* L. [Ginkgoaceae; Ginkgo],10 mg.	Y—Prepared according to the National Food and Drug Administration National Drug Standards	N	Activating blood and removing stasis, restoring pulse and alleviating pain
Li, (2017)	Yiqihuayu capsule	[Tangshan Hospital of Traditional Chinese Medicine]	*Astragalus mongholicus* Bunge [Fabaceae; Astragalus]; *Pseudostellaria* Pax [Caryophyllaceae; Pseudostellaria]; *Conioselinum anthriscoides* ‘Chuanxiong’ [Apiaceae; Conioselinum]; Pheretima [Siliquariidae; Pheretima]; *Paeonia rubra* Steud. [Paeoniaceae; Paeonia]; and *Spatholobus suberectus* Dunn [Fabaceae; Spatholobus].	N	N	Warming kidney and replenishing *qi*, activating blood, and removing stasis
Yang et al. (2012)	Tongxinluo capsule	[Shijiazhuang Yiling Pharmaceutical Co., Ltd.]	*Panax ginseng* C.A.Mey. [Araliaceae; Panax]; *Whitmania pigra* Whitman [Hirudinidae; Hirudo]; scorpion [Buthidae; Scorpio]; ground beetle; *Scolopendra subspinipes* [Scolopendridae; centipede]; *Cryptotympana atrata* Fabricius [Cicadidae; Periostracum Cicadae]; *Paeonia rubra* Steud. [Paeoniaceae; Paeonia]; *Dalbergia odorifera* T.C.Chen [Fabaceae; Dalbergia]; *Pistacia lentiscus* L. [Anacardiaceae; Pistacia]; *Santalum album* L. [Santalaceae; Santalum]; *Ziziphus jujuba* Mill. [Rhamnaceae; Ziziphus]; and *Dipterocarpus turbinatus* C.F.Gaertn. [Dipterocarpaceae; Dipterocarpus].	Y—Prepared according to People’s Republic of China Pharmacopoeia, 2020	Y—HPLC	Replenishing *qi* and activating blood, removing stasis, and restoring pulse
Wu and Hu, (2019)	Musk tongxin dropping pills	[Inner Mongolia Kangenbei Pharmaceutical Co. Ltd. Shenglong Branch]	*Panax ginseng* C.A.Mey. [Araliaceae; Panax]; artificial *Moschus*; *Salvia miltiorrhiza* Bunge [Labiatae; Salvia]; *Dipterocarpus turbinatus* C.F.Gaertn. [Dipterocarpaceae; Dipterocarpus]; toad [Bufonidae; Toad Venom]; Ursidae [Ursidae; Bear gall powder]; and artificial bezoar.	Y—Prepared according to People’s Republic of China Pharmacopoeia, 2020	Y—HPLC	Inducing aromatic, replenishing *qi* and restoring pulse, activating blood, removing stasis, and alleviating pain
Wu et al. (2021)	Musk tongxin dropping pills	[Inner Mongolia Kangenbei Pharmaceutical Co. Ltd. Shenglong Branch]	*Panax ginseng* C.A.Mey. [Araliaceae; Panax]; Artificial *Moschus*; *Salvia miltiorrhiza* Bunge [Labiatae; Salvia]; *Dipterocarpus turbinatus* C.F.Gaertn. [Dipterocarpaceae; Dipterocarpus]; toad [Bufonidae; Toad Venom]; Ursidae [Ursidae; Bear gall powder]; and artificial bezoar.	Y—Prepared according to People’s Republic of China Pharmacopoeia, 2020	Y—HPLC	Inducing aromatic, replenishing *qi* and restoring pulse, activating blood, removing stasis, and alleviating pain
Zou, (2019)	Heart-protecting musk pill	[Shanghai Hehuang Pharmaceutical Co. Ltd.]	*Moschus* [Cervidae; Moschus]; *Panax ginseng* C.A.Mey. [Araliaceae; Panax]; oriental sweetgum [Hamamelidaceae; Styralyl propionate]; Bovis Calculus [Bovine; *Calculus bovis*]; *Cinnamomum cassia* Siebold [Lauraceae; Cinnamomum]; and toad [Bufonidae; toad venom].	Y—Prepared according to People’s Republic of China Pharmacopoeia, 2005	Y—HPLC	Replenishing *qi* and restoring pulse, activating blood, and removing stasis
Yang and Liu, (2020)	Sanwei sandalwood capsule	[Inner Mongolia Kemeng Pharmaceutical Co. Ltd.]	*Santalum album* L. [Santalaceae; Santalum]; *Choerospondias axillaris* (Roxb.) B.L.Burtt and A.W.Hill [Anacardiaceae; Choerospondias]; and *Myristica fragrans* Houtt. [Myristicaceae; Myristica].	Y—Prepared according to the National Food and Drug Administration National Drug Standards.	N	Clearing heat
Xu et al. (2019)	Compound Danshen dripping pills	[Tianjin Tasly Pride Pharmaceutical Co., Ltd.]	*Salvia miltiorrhiza* Bunge [Labiatae; Salvia], 90g; *Panax notoginseng* (Burkill) F.H.Chen [Araliaceae; Panax], 17.6g; and *Dipterocarpus turbinatus* C.F.Gaertn. [Dipterocarpaceae; Dipterocarpus], 1 g.	Y—Prepared according to People’s Republic of China Pharmacopoeia, 2020	Y—HPLC	Activating blood and removing stasis, promoting *qi*, and alleviating pain
Fan. (2017)	Qiliqiangxin granules	[Shijiazhuang Yiling Pharmaceutical Co., Ltd.]	*Astragalus mongholicus* Bunge [Fabaceae; Astragalus], 450g; *Panax ginseng* C.A.Mey. [Araliaceae; Panax], 225g; *Aconitum carmichaelii* Debeaux [Ranunculaceae; Aconitum], 112.5g; *Salvia miltiorrhiza* Bunge [Lamiaceae; Salvia], 225g; *Lepidium apetalum* Willd. [Brassicaceae; Lepidium], 150g; *Alisma plantago-aquatica* L. [Alismataceae; Alisma], 225g; *Polygonatum odoratum* (Mill.) Druce [Asparagaceae; Polygonatum], 75g; *Cinnamomum cassia* Siebold [Lauraceae; Cinnamomum], 90g; *Carthamus tinctorius* L. [Asteraceae; Carthamus], 90g; *Periploca sepium* Bunge [Apocynaceae; Periploca], 180g; and *Citrus reticulata* Blanco [Rutaceae; Citrus], 75 g.	Y—Prepared according to People’s Republic of China Pharmacopoeia, 2020	Y—HPLC	Replenishing *qi* and restoring *yang*, and restoring pulse and cardiotonic
Zhang et al. (2017)	Qiliqiangxin granules	[Shijiazhuang Yiling Pharmaceutical Co., Ltd.]	*Astragalus mongholicus* Bunge [Fabaceae; Astragalus], 450g; *Panax ginseng* C.A.Mey. [Araliaceae; Panax], 225g; *Aconitum carmichaelii* Debeaux [Ranunculaceae; Aconitum], 112.5g; *Salvia miltiorrhiza* Bunge [Lamiaceae; Salvia], 225g; *Lepidium apetalum* Willd. [Brassicaceae; Lepidium], 150g; *Alisma plantago-aquatica* L. [Alismataceae; Alisma], 225g; *Polygonatum odoratum* (Mill.) Druce [Asparagaceae; Polygonatum], 75g; *Cinnamomum cassia* Siebold [Lauraceae; Cinnamomum], 90g; *Carthamus tinctorius* L. [Asteraceae; Carthamus], 90g; *Periploca sepium* Bunge [Apocynaceae; Periploca], 180g; and *Citrus reticulata* Blanco [Rutaceae; Citrus], 75 g.	Y—Prepared according to People’s Republic of China Pharmacopoeia, 2020	Y—HPLC	Replenishing qi and restoring yang, and restoring pulse and cardiotonic
Zheng, (2013)	Fumai granule	Prepared by Huang C.L.	*Panax ginseng* C.A.Mey. [Araliaceae; Panax]; *Ophiopogon japonicus* (Thunb.) Ker Gawl. [Asparagaceae; Ophiopogon]; and *Schisandra chinensis* (Turcz.) Baill. [Schisandraceae; Schisandra].	N	N	Replenishing *qi*, nourishing *yin*, and restoring pulse
Peng et al. (2020)	Jianxin Pinglv pills	[Shenzhen Traditional Chinese Medicine Hospital]	*Astragalus mongholicus* Bunge [Fabaceae; Astragalus]; *Pseudostellaria* Pax [Caryophyllaceae; Pseudostellaria]; *Ophiopogon japonicus* (Thunb.) Ker Gawl. [Asparagaceae; Ophiopogon]; Caulis Bambusae in Taeniam [Poaceae; Phyllostachys]; *Pinellia ternata* (Thunb.) Makino [Araceae; Pinellia]; *Citrus reticulata* Blanco [Rutaceae; Citrus]; *Atractylodes macrocephala* Koidz. [Asteraceae; Atractylodes]; *Salvia miltiorrhiza* Bunge [Lamiaceae; Salvia]; *Panax notoginseng* (Burkill) F.H.Chen [Araliaceae; Panax]; and *Ziziphus jujuba* Mill. [Rhamnaceae; Ziziphus].	N	N	Replenishing *qi* and nourishing *yin*, resolving phlegm and removing stasis, calming heart, and tranquilizing mind
Hu, (2021)	Kidney-*qi*-tonifying pill	[Beijing Tongrentang Science and Technology Development Co., Ltd. pharmaceutical factory]	*Rehmannia glutinosa* (Gaertn.) DC. [Orobanchaceae; Rehmannia], 108g; *Dioscorea polystachya* Turcz. [Dioscoreaceae; Dioscorea], 27g; *Cornus officinalis* Siebold and Zucc. [Cornaceae; Cornus], 27g; *Poria cocos* (Schw.) Wolf [Polyporaceae; Poria], 78g; Paeonia × suffruticosa Andrews [Paeoniaceae; Paeonia], 27g; *Alisma plantago-aquatica* L. [Alismataceae Alisma], 27g; *Cinnamomum cassia* Siebold [Lauraceae; Cinnamomum], 27g; *Aconitum carmichaelii* Debeaux [Ranunculaceae; Aconitum], 4.5g; *Achyranthes bidentata* Blume [Amaranthaceae; Achyranthes], 27g; and *Plantago asiatica* L. [Plantaginaceae; Plantago], 27 g.	Y—Prepared according to People’s Republic of China Pharmacopoeia, 1963	N	Warming kidney and restoring *yang*
Meng et al. (2021)	Compound Danshen dripping pills	[Tianjin Tasly Pride Pharmaceutical Co., Ltd.]	*Salvia miltiorrhiza* Bunge [Labiatae; Salvia], 90g; *Panax notoginseng* (Burkill) F.H.Chen [Araliaceae; Panax], 17.6 g; and *Dipterocarpus turbinatus* C.F.Gaertn. [Dipterocarpaceae; Dipterocarpus], 1 g.	Y—Prepared according to People’s Republic of China Pharmacopoeia, 2020	Y—HPLC	Activating blood and removing stasis, promoting *qi*, and alleviating pain
Ji et al. (2021)	Buxinqi oral solution	[Hubei Furen Jinshen Pharmaceutical Co. Ltd.]	*Astragalus mongholicus* Bunge [Fabaceae; Astragalus], 500g; *Panax ginseng* C.A.Mey. [Araliaceae; Panax], 100g; *Acorus tatarinowii* [Araceae; Acorus], 333g; and *Allium macrostemon* Bunge. [Amaryllidaceae; Allium], 200 g.	Y—Prepared according to People’s Republic of China Pharmacopoeia, 2020	Y—HPLC	Tonifying heart, replenishing and regulating *qi*, and alleviating pain
Liu, (2014)	Wenxin granule	[Shandong Buchang Pharmaceutical Co. Ltd.]	*Codonopsis pilosula* (Franch.) Nannf. [Campanulaceae; Codonopsis], 300g; *Panax notoginseng* (Burkill) F.H.Chen [Araliaceae; Panax], 60g; *Nardostachys jatamansi* (D.Don) DC [Caprifoliaceae; Nardostachys], 200g; Ambrum, 40g; *Polygonatum odoratum* (Mill.) Druce [Asparagaceae; Polygonatum], 400 g.	Y—Prepared according to People’s Republic of China Pharmacopoeia, 2020	Y—HPLC	Tonifying blood and replenishing *qi*, activating blood and removing stasis, calming heart, and tranquilizing mind
Xie et al. (2019)	Qishen Yiqi Droplet	[Tianjin Tasly Pride Pharmaceutical Co., Ltd.]	*Astragalus mongholicus* Bunge [Fabaceae; Astragalus], 1800g; *Salvia miltiorrhiza* Bunge [Labiatae; Salvia], 900; *Dalbergia odorifera* T.C.Chen [Fabaceae; Dalbergia], 12g; and *Panax notoginseng* (Burkill) F.H.Chen [Araliaceae; Panax], 180 g.	Y—Prepared according to People’s Republic of China Pharmacopoeia, 2020	Y—HPLC	Replenishing *qi* and restoring pulse, activating blood, and alleviating pain
Zhang and Gou, (2021)	Qishen Yiqi Droplet	[Tianjin Tasly Pride Pharmaceutical Co., Ltd.]	*Astragalus mongholicus* Bunge [Fabaceae; Astragalus], 1800 g; *Salvia miltiorrhiza* Bunge [Labiatae; Salvia], 900; *Dalbergia odorifera* T.C.Chen [Fabaceae; Dalbergia], 12 g; and *Panax notoginseng* (Burkill) F.H.Chen [Araliaceae; Panax], 180 g.	Y—Prepared according to People’s Republic of China Pharmacopoeia, 2020	Y—HPLC	Replenishing *qi* and restoring pulse, activating blood, and alleviating pain

According to the efficacy noted in the original text of the included studies, the CPMs in this study were categorized as *qi*-replenishing Chinese patent medicine (QR-CPM); blood-activating and stasis-removing CPM (BASR-CPM); heat-clearing CPM (HC-CPM); kidney-warming and *yang*-restoring CPM (KWYR-CPM); and *qi*-replenishing, blood-activating, and stasis-removing CPM (QR&BASR-CPM).

### Summary of quality assessment

**FIGURE 2:**
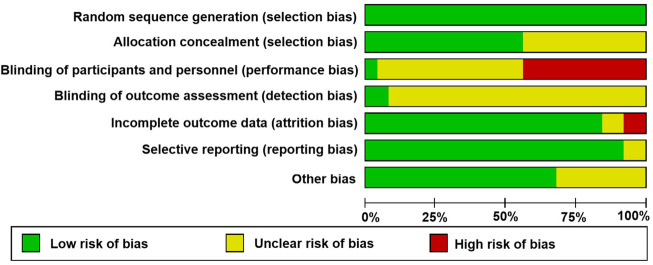
Risk of bias.

**FIGURE 3:**
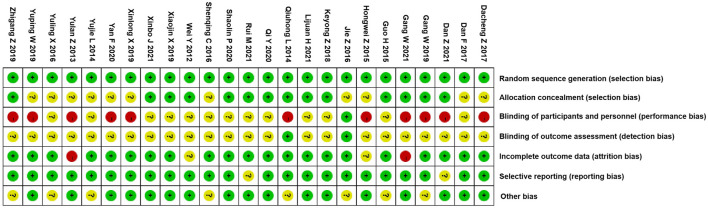
Summary of risk of bias based on selection bias.

The detailed risk of bias is shown in Figure 2 according to assessment with the Cochrane Collaboration tool. A random sequence was generated in all the studies, suggesting the risk of bias in randomization was low. Among them, 14 were rated as a low risk of bias and 11 had unclear information about the methods used to conceal the allocation; therefore, we considered that the risk of bias was unclear for the domain of allocation concealment. Regarding the blinding of participants and personnel, high risk was observed in 11 RCTs; a large proportion of studies had an unclear risk of detection bias. As for incomplete outcomes, there was a low risk of bias because most studies (*n* = 20) reported complete data. Only two had unclear risks regarding selective reporting bias, and seven had unclear risks of other bias. Overall, 25 trials were deemed as unclear bias risk, which showed that the certainty of their evidence was moderate. For a summary of quality assessment, see Figure 3.

### Network meta-analysis (NMA) results

In network maps, the center of WM was compared to eight CPM-related treatment regimens in IG, among which a combined regimen was denoted with “+” signs. The thickness of the line means the number of trials, and the size of the circle indicates the number of patients included. Detailed information for each treatment regimen is presented in Table 3.

**TABLE 3:** Detailed information for each treatment regimen of all outcomes.

Treatment regimen	No. of trials	No. of patients
Clinical efficacy	19	1966
WM	19	982
BASR-CPM	1	64
QR&BASR-CPM	3	197
QR-CPM + WM	8	349
HC-CPM + WM	1	43
BASR-CPM + WM	1	40
QR&BASR-CPM + WM	5	291
**LVEF**	**21**	**1903**
WM	21	950
BASR-CPM	2	110
QR&BASR-CPM	2	151
QR-CPM + WM	8	323
HC-CPM + WM	1	43
BASR-CPM + WM	4	164
QR&BASR-CPM + WM	3	126
KWYR-CPM + THSWT + WM	1	36
**LVEDD**	**14**	**1278**
WM	14	639
QR&BASR-CPM	2	151
QR-CPM + WM	6	252
BASR-CPM + WM	2	74
QR&BASR-CPM + WM	3	126
KWYR-CPM + THSWT + WM	1	36
**LVESD**	**9**	**821**
WM	9	410
QR&BASR-CPM	1	86
QR-CPM + WM	4	159
BASR-CPM + WM	1	40
QR&BASR-CPM + WM	3	126
**LVMI**	**7**	**714**
WM	7	362
QR-CPM + WM	1	40
QR&BASR-CPM	2	151
BASR-CPM + WM	2	84
QR&BASR-CPM + WM	1	50
KWYR-CPM + THSWT + WM	1	36
**CRP**	**7**	**689**
WM	7	338
BASR-CPM	2	110
BASR-CPM + WM	2	74
QR-CPM + WM	2	71
QR&BASR-CPM	1	86
**NT-proBNP**	**6**	**609**
WM	6	305
BASR-CPM	1	64
QR&BASR-CPM	1	65
QR-CPM + WM	2	70
BASR-CPM + WM	1	34
QR&BASR-CPM + WM	1	71
**6-MWT**	**6**	**615**
WM	6	308
BASR-CPM	1	64
QR&BASR-CPM	1	65
QR-CPM + WM	3	142
KWYR-CPM + THSWT + WM	1	36

Note: Specific treatment regimen information is commented upon as in Table 1.

The bold values indicated outcome indicators in the study.

**FIGURE 4:**
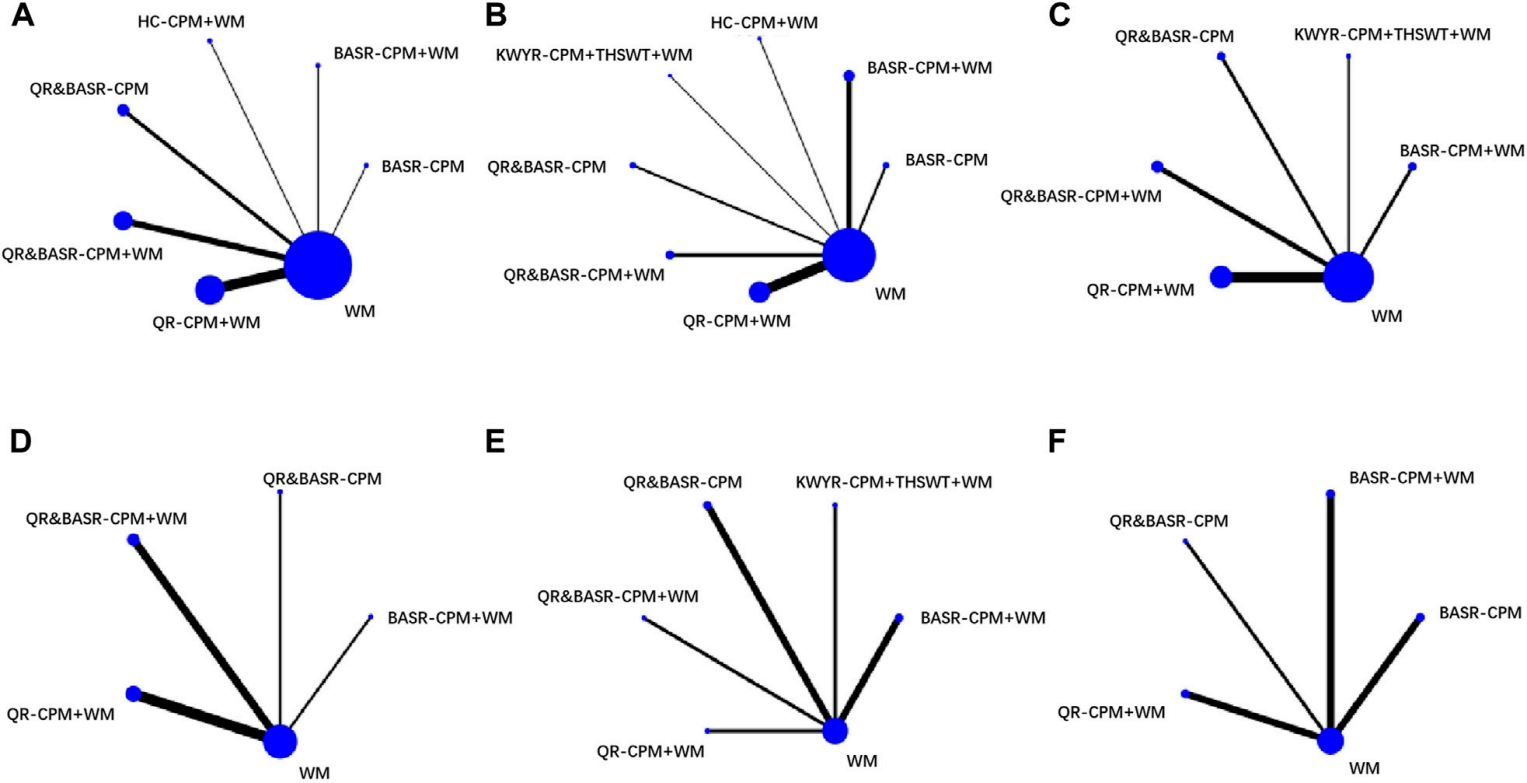
Network map. Notes: **(A)**: Clinical Efficacy; **(B)**: LVEF; **(C)**: LVEDD; **(D)**: LVESD; **(E)**: LVMI; **(F)**: CRP; QR-CPM: Qi-replenishing Chinese patent medicine; BASR-CPM: Blood-activating and stasis removing Chinese patent medicine; HC-CPM: Heat-clearing Chinese patent medicine, KWYR-CPM: Kidney warming and yang-restoring Chinese patent medicine; QR&BASR-CPM: Qi-replenishing, blood-activating, and stasis-removing Chinese patent medicine; THSWT: TaohongSiwu decoction; WM: Western Medicine. The “+” sign indicated combined regimens.

In the network map for primary outcomes, WM as a center point is compared by six CPM-related treatment regimens of IG, among which the circle of QR-CPM + WM had the largest number of included patients in IG and the connection line between QR-CPM + WM and WM has the largest quantity of included trials (Figure 4A). In the network map for secondary outcomes, the point and line results regarding LVEF and LVEDD of echocardiographic indices were the same as the primary outcome (Figures 4B, C). Other secondary outcomes including echocardiographic indices (LVESD and LVMI) are shown in Figures 4D and E, cardiac biomarkers (CRP and NT-proBNP) which show CRP in Figure 4F, NT-proBNP in Supplementary Figure S1A, and functional exercise capacity (6-MWT) in Supplementary Figure S1B. The overall network relationship was centered on WM, which had no closed ring.

### Difference in mean changes in primary clinical efficacy

Clinical efficiency was useful for planning future clinical trials on cardiac hypertrophy treatment and predicting the impact of CPM-related treatment regimens.

Azzimondi et al. (1997) Some 19 studies (Zheng, 2013; Liu, 2014; Han and Shen, 2015; Xun, 2016; Zhang et al., 2016; Fan, 2017; Li, 2017; Zhang et al., 2017; Zhou et al., 2018; Wang et al., 2019; Wu and Hu, 2019; Xu et al., 2019; Zou, 2019; Fan et al., 2020; Peng et al., 2020; Yang and Liu, 2020; Ji et al., 2021; Wu et al., 2021; Zhang and Gou, 2021) involving 1,966 patients comprised WM in CG, and six CPM-related treatment regimens including QR-CPM + WM, BASR-CPM, BASR-CPM + WM, QR&BASR-CPM, QR&BASR-CPM + WM, and HC-CPM + WM in IG were measured for clinical efficacy.

The pairwise comparison of clinical efficacy was evaluated by OR and 95% CI for each treatment regimen, which is shown in a league table (Table 4) and forest map (Supplementary Figure S2A). First, BASR-CPM + WM was more significant for improving clinical efficacy than any other treatment regimen, especially over WM (OR = 8.27; 95%CI = 0.97,70.73)—however, there was no statistical difference. Second, all CPM-related treatment regimens in IG were superior to CG in clinical efficacy, which had significant differences expect for BASR-CPM + WM. The pairwise comparisons of all treatment regimens in IG had no differences.

**TABLE 4:** Results of network meta-analysis for clinical efficacy.

BASR-CPM+WM
1.33 (0.09, 19.20)	HC-CPM+WM					
1.57 (0.17, 14.76)	1.18 (0.21, 6.53)	QR&BASR-CPM+WM				
2.14 (0.23, 19.84)	1.61 (0.30, 8.74)	1.37 (0.57, 3.30)	QR&BASR-CPM			
2.35 (0.26, 20.96)	1.77 (0.34, 9.12)	1.50 (0.69, 3.27)	1.10 (0.53, 2.27)	QR-CPM+WM		
2.80 (0.26, 30.09)	2.10 (0.32, 13.84)	1.78 (0.53, 5.99)	1.31 (0.40, 4.25)	1.19 (0.39, 3.59)	BASR-CPM	
8.27 (0.97, 70.73)	6.21 (1.27, 30.34)	5.28 (2.75, 10.14)	3.86 (2.14, 6.98)	3.52 (2.30, 5.38)	2.96 (1.07, 8.21)	WM

Notes: The bold values indicated that the pairwise comparison between treatment regimens are statistical significance differences (*p* < 0.05). The treatment regimens is the same as the note of Figure 4.

According to the SUCRA probability in improving clinical efficacy, BASR-CPM + WM had the highest probability of being the best treatment regimen out of the seven (Supplementary Figure S3A).

### Difference in mean changes in Echocardiographic indices

Changes in echocardiographic indices can reflect the morphological features of progressive cardiac remodeling (Cheng et al., 2010).

### Left ventricular ejection fraction (LVEF)

All data from the 21 studies (Yang et al., 2012; Zheng, 2013; Han and Shen, 2015; Cui et al., 2016; Zhang et al., 2016; Fan, 2017; Li, 2017; Zhang et al., 2017; Wu and Hu, 2019; Xie et al., 2019; Xu et al., 2019; Zheng et al., 2019; Zou, 2019; Fan et al., 2020; Peng et al., 2020; Yang and Liu, 2020; Hu, 2021; Ji et al., 2021; Meng et al., 2021; Wu et al., 2021; Zhang and Gou, 2021) were analyzed—they consisted of 1,903 patients with WM in the CG and seven CPM-related treatment regimens in the IG (QR&BASR-CPM + WM, BASR-CPM, BASR-CPM + WM, KWYR-CPM + THSWT + WM, QR-CPM + WM, HC-CPM + WM, and QR&BASR-CPM).

The pairwise comparison of each treatment regimen in IG and CG is shown in Table 5 and Supplementary Figure S2B. The results show that BASR-CPM was the most effective treatment regimen in increasing the level of LVEF in the IG. In addition, QR&BASR-CPM + WM (SMD = 1.67; 95%CI = 0.48, 2.86), BASR-CPM + WM (SMD = 1.54; 95%CI = 0.50, 2.59), and QR-CPM + WM (SMD = 0.87, 95%CI = 0.14.1.59) also had significant improvement compared to WM. All mean changes in pairwise comparison had no differences within the IG.

**TABLE 5:** Results of network meta-analysis for LVEF.

BASR-CPM
0.24 (-1.64, 2.11)	QR&BASR-CPM+WM						
0.36 (-1.41, 2.13)	0.12 (-1.45, 1.69)	BASR-CPM+WM					
0.46 (-2.05, 2.97)	0.23 (-2.14, 2.60)	0.10 (-2.19, 2.40)	KWYR-CPM+THSWT+WM				
1.13 (-1.36, 3.63)	0.90 (-1.46, 3.25)	0.77 (-1.50, 3.05)	0.67 (-2.21, 3.56)	HC-CPM+WM			
1.04 (-0.58, 2.65)	0.80 (-0.59, 2.19)	0.68 (-0.58, 1.93)	0.57 (-1.60, 2.75)	-0.10 (-2.25, 2.06)	QR-CPM+WM		
1.16 (-0.87, 3.19)	0.92 (-0.93, 2.77)	0.80 (-0.96, 2.55)	0.69 (-1.80,3.19)	0.02 (-2.46,2.50)	0.12 (-1.48,1.71)	QR&BASR-CPM	
1.90 (0.46, 3.35)	1.67 (0.48, 2.86)	1.54 (0.52, 2.57)	1.44 (-0.61, 3.49)	0.77 (-1.26, 2.80)	0.87 (0.14, 1.59)	0.75 (-0.68, 2.17)	WM

Notes: The bold values indicated that the pairwise comparison between treatment regimens are statistical significance differences (*p* < 0.05). The treatment regimens is the same as the note of Figure 4.

The SUCRA for each regimen indicated that the use of BASR-CPM (78.2%) had the highest probability of being the best option for effectively improving the level of LVEF among the eight types of treatment regimens (Supplementary Figure S3B).

### Left ventricular end-diastolic dimension (LVEDD)

Out of 25 RCTs, 14 (Yang et al., 2012; Fan, 2017; Zhang et al., 2017; Wu and Hu, 2019; Xie et al., 2019; Xu et al., 2019; Zheng et al., 2019; Zou, 2019; Peng et al., 2020; Hu, 2021; Ji et al., 2021; Meng et al., 2021; Wu et al., 2021; Zhang and Gou, 2021) were analyzed, consisting of 1,278 patients undergoing five IG treatment regimens—QR-CPM + WM, BASR-CPM + WM, QR&BASR-CPM, QR&BASR-CPM + WM, and KWYR-CPM + THSWT + WM—and WM in the CG.

The pairwise comparison outcome of LVEDD is presented in Table 6 and Supplementary Figure S2C. Compared to WM, KWYR-CPM + THSWT + WM was superior to other treatment regimens in reducing LVEDD. Meanwhile, compared with WM, QR&BASR-CPM + WM (SMD = −1.54; 95%CI = −2.49, −0.60) and QR-CPM + WM (SMD = −0.76; 95%CI = −1.41, −0.11) were also significantly associated with a reduction in LVEDD. When the pairwise comparison was made between treatment regimens in the IG, all mean changes showed no significant differences within it.

**TABLE 6:** Results of network meta-analysis for LVEDD.

KWYR-CPM+THSWT+WM
-0.30 (-2.17, 1.58)	QR&BASR-CPM+WM				
-0.75 (-2.73, 1.23)	-0.45 (-1.93, 1.03)	BASR-CPM+WM			
-1.08 (-2.83, 0.67)	-0.79 (-1.93, 0.36)	-0.34 (-1.65, 0.97)	QR-CPM+WM		
-1.32 (-3.28, 0.64)	-1.02 (-2.47, 0.43)	-0.57 (-2.16, 1.01)	-0.24 (-1.52, 1.04)	QR&BASR-CPM	
-1.84 (-3.46, -0.22)	-1.54 (-2.49, -0.60)	-1.09 (-2.23, 0.04)	-0.76 (-1.41, -0.11)	-0.52 (-1.62, 0.58)	WM

Notes: The bold values indicated that the pairwise comparison between treatment regimens are statistical significance differences (*p* < 0.05). The treatment regimens is the same as the note of Figure 4.

Meanwhile, the SUCRA result showed that KWYR-CPM + THSWT + WM (82.7%) had the highest probability of being ranked first in reducing the level of LVEDD, followed by others in the IG (Supplementary Figure S3C).

### Left ventricular end-systolic dimension (LVESD)

Nine trials (Yang et al., 2012; Fan, 2017; Zhang et al., 2017; Wu and Hu, 2019; Xie et al., 2019; Xu et al., 2019; Zheng et al., 2019; Zou, 2019; Zhang and Gou, 2021) of LVESD involved 821 patients with four CPM-related treatment regimens and one WM. QR&BASR-CPM + WM (SMD = −2.35; 95%CI = −3.09,−1.62) was the most effective treatment regimen in decreasing LVESD with significant differences expect for BASR-CPM (Table 7; Supplementary Figure S2D).

**TABLE 7:** Results of network meta-analysis for LVESD.

QR&BASR-CPM+WM
-1.25 (-2.66, 0.15)	BASR-CPM+WM			
-1.67 (-2.62, -0.72)	-0.41 (-1.76, 0.93)	QR-CPM+WM		
-2.15 (-3.51, -0.79)	-0.90 (-2.56, 0.76)	-0.48 (-1.78, 0.81)	QR&BASR-CPM	
-2.35 (-3.09, -1.62)	-1.10 (-2.30, 0.10)	-0.69 (-1.29, -0.09)	-0.20 (-1.35, 0.94)	WM

Notes: The bold values indicated that the pairwise comparison between treatment regimens are statistical significance differences (*p* < 0.05). The treatment regimens is the same as the note of Figure 4.

SUCRA indicated QR&BASR-CPM + WM to be ranked first and considered the best option for reducing the level of LVESD (Supplementary Figure S3D).

### Left ventricular mass index (LVMI)

LVMI changes in patients were reported in seven studies (Liu, 2014; Cui et al., 2016; Wu and Hu, 2019; Xie et al., 2019; Hu, 2021; Meng et al., 2021; Wu et al., 2021) involving five treatment regimens in IG plus a WM in CG. QR&BASR-CPM + WM and BASR-CPM + WM were considered effective treatment regimens for reducing the level of LVM with significant differences (Table 8; Supplementary Figure S2E).

**TABLE 8:** Results of network meta-analysis for LVMI.

QR&BASR-CPM+WM
-0.59 (-2.06, 0.87)	BASR-CPM+WM				
-1.05 (-2.49, 0.39)	-0.45 (-1.63, 0.72)	QR&BASR-CPM			
-1.10 (-2.79, 0.59)	-0.50 (-1.97, 0.96)	-0.05 (-1.49, 1.40)	KWYR-CPM+THSWT+WM		
-1.35 (-3.03, 0.32)	-0.76 (-2.22, 0.70)	-0.31 (-1.74, 1.13)	-0.26 (-1.94, 1.43)	QR-CPM+WM	
-1.73 (-2.92, -0.54)	-1.14 (-1.99, -0.29)	-0.68 (-1.49, 0.13)	-0.63 (-1.83, 0.56)	-0.37 (-1.56, 0.81)	WM

Notes: The bold values indicated that the pairwise comparison between treatment regimens are statistical significance differences (*p* < 0.05). The treatment regimens is the same as the note of Figure 4.

On the basis of SUCRA results, QR&BASR-CPM + WM (90.9%) is most probably the best treatment regimen for reducing LVMI (Supplementary Figure S3E).

### Difference in mean changes in cardiac biomarkers

Cardiac biomarkers suggest that their measurement can be used for preclinical diagnosis of left ventricular hypertrophy (LVH) (Koycheva et al., 2016).

### C-reactive protein (CRP)

Seven trials (Zheng, 2013; Zhang et al., 2016; Fan, 2017; Li, 2017; Wu and Hu, 2019; Fan et al., 2020; Meng et al., 2021) with CRP included 679 patients with four CPM-related treatment regimens in IG and a WM in CG.

There was no significant difference in CRP between the two treatment regimens related to BASR: BASR-CPM and BASR-CPM + WM (Table 9; Supplementary Figure S2F).

**TABLE 9:** Results of network meta-analysis for CRP.

BASR-CPM
-0.49 (-1.51, 0.53)	BASR-CPM+WM			
-0.79 (-1.99, 0.40)	-0.30 (-1.51, 0.90)	QR&BASR-CPM		
-0.98 (-2.00, 0.03)	-0.49 (-1.52, 0.54)	-0.19 (-1.39, 1.01)	QR-CPM+WM	
-1.31 (-2.02, -0.60)	-0.82 (-1.55, -0.09)	-0.52 (-1.48, 0.44)	-0.33 (-1.05, 0.40)	WM

Notes: The bold values indicated that the pairwise comparison between treatment regimens are statistical significance differences (*p* < 0.05). The treatment regimens is the same as the note of Figure 4.

By SUCRA, BASR-CPM (92.3%) had the highest probability of being ranked first in reducing CRP (Supplementary Figure S3F).

### N-terminal proBNP (NT-proBNP)

The pairwise comparison in NT-proBNP manifesting among each treatment regimen showed no differences in all included studies (Table 10; Supplementary Figure S2G). Therefore, the results for NT-proBNP do not allow clear conclusions to be drawn.

**TABLE 10:** Results of network meta-analysis for NT-proBNP.

BASR-CPM
-0.28 (-4.22, 3.65)	QR-CPM+WM				
-0.31 (-4.84, 4.22)	-0.03 (-3.95, 3.90)	QR&BASR-CPM+WM			
-0.57 (-5.11, 3.98)	-0.28 (-4.23, 3.66)	-0.26 (-4.80, 4.28)	BASR-CPM+WM		
-2.04 (-5.96, 1.88)	-1.75 (-4.97, 1.46)	-1.73 (-5.65, 2.19)	-1.47 (-5.41, 2.46)	QR&BASR-CPM	
-2.13 (-5.33, 1.07)	-1.85 (-4.13, 0.44)	-1.82 (-5.02, 1.38)	-1.56 (-4.78, 1.66)	-0.09 (-2.35, 2.17)	WM

### Difference in mean changes in functional exercise capacity

#### Six-minute walk test (6-MWT)

Functional capacity was measured with 6-MWT, a well-tolerated, practical, and useful tool with worldwide recommendations for the cardiorespiratory domain (Hojan et al., 2020). There is no statistical significance in the pairwise comparison among all treatment regimens (Table 11; Supplementary Figure S2H **)**. Therefore, the results for 6-MWT do not enable clear conclusions to be drawn.

**TABLE 11:** Results of network meta-analysis for 6-MWT.

QR-CPM+WM
0.43 (-3.77, 4.62)	QR&BASR-CPM			
0.57 (-3.64, 4.78)	0.14 (-5.00,5.29)	KWYR-CPM+THSWT+WM		
0.91 (-3.29, 5.10)	0.48 (-4.65, 5.61)	0.34 (-4.81, 5.48)	BASR-CPM	
1.68 (-0.42, 3.79)	1.25 (-2.37, 4.88)	1.11 (-2.53, 4.75)	0.77 (-2.85, 4.40)	WM

Using SUCRA , the probability rank information on all treatment regimens for each outcome is summarized in Table 12.

**TABLE 12:** All treatment regimen information on each outcome.

Treatment regimen outcome	Clinical efficacy	LVEF	LVEDD	LVESD	LVMI	CRP	NT-proBNP	6-MWT
WM	7	8	6	5	6	5	6	5
BASR-CPM	6	1	NA	NA	NA	1	1	4
QR&BASR-CPM	4	7	5	4	3	3	5	2
QR-CPM + WM	5	6	4	3	5	4	2	1
BASR-CPM + WM	1	3	3	2	2	2	4	NA
QR&BASR-CPM + WM	3	2	2	1	1	NA	3	NA
KWYR-CPM + THSWT + WM	NA	4	1	NA	4	NA	NA	3
HC-CPM + WM	2	5	NA	NA	NA	NA	NA	NA

Note: Specific treatment regimen information is commented upon as in Table 1.

#### Risk of publication bias

**FIGURE 5:**
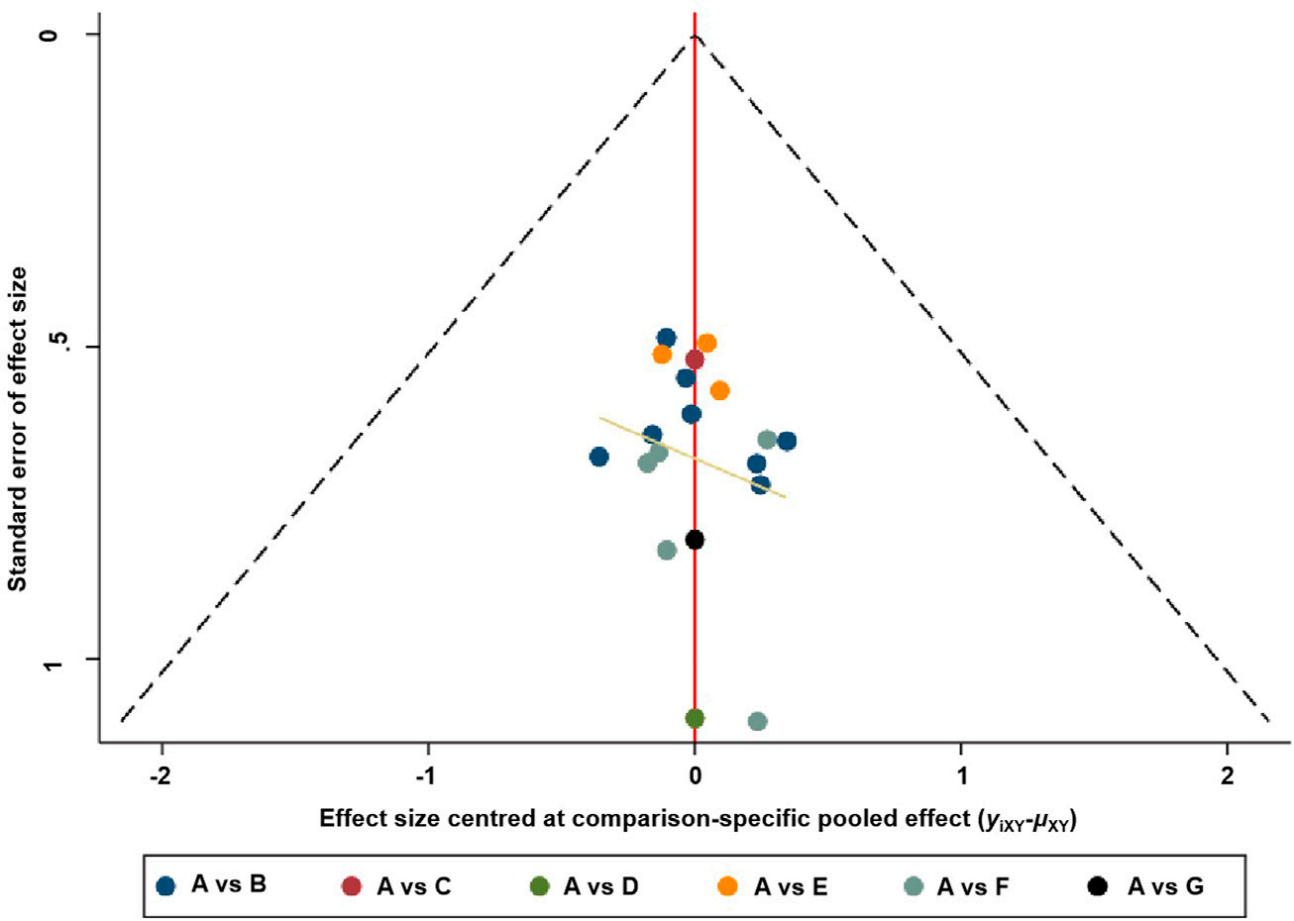
Funnel plot for clinical efficacy.

The risk of publication bias could be assessed by a funnel plot. The funnel plot about the primary outcome of clinical efficacy is shown in Figure 5, and the secondary outcomes with LVEF and LVEDD are shown in Supplementary Figure S4A, B **—**funnel plots were not feasible for those including fewer than 10 studies. According to the funnel plot of the primary outcome, all trials were basically distributed on both sides of the middle, and the left and right distribution were roughly symmetrical; this indicated no strong evidence of publication bias in all the trials for the primary outcome. The results for other funnel plots also indicated no strong publication bias.

## Discussion

With increasing clinical trials into combining traditional and Western medicines in the treatment of myocardial hypertrophy, this study conducted a NMA to compare for clinical efficacy, echocardiographic indices, cardiac biomarkers, and functional capacity for each CPM-related treatment regimen used for patients with cardiac hypertrophy. It provided scientific and meaningful evidence for precision medicine in clinical settings. The results of pairwise comparison of different treatment regimens in the present network meta-analysis showed that BASR-CPM + WM (OR = 8.27; 95%CI = 0.97.70.73) might be the optimum selection, being ranked first for improving clinical efficacy over other treatment regimens. It is worth mentioning that QR&BASR-CPM + WM showed the highest effectiveness in echocardiographic indices such as reducing LVESD (SMD = −2.35; 95%CI = −3.09, −1.62) and LVMI (SMD = −1.73; 95%CI = −2.92, -0.54). For included studies in this meta-analysis, there were no overall obvious publication bias or small-study effects.

BASR-CPM + WM, as mainly used for treating cardiovascular diseases, had the highest clinical efficacy (OR = 8.27; 95%CI = 0.97, 70.73) in this study; the function of blood activation and stasis removal could unblock and activate blood vessels by relieving blockages, resisting myocardial ischemia and inhibiting platelet aggregation and anticoagulant and antithrombotic formation, leading to improved cardiovascular blood supply (Zhou et al., 2017; Liu et al., 2019). The components of BASR-CPM for treating cardiovascular and cerebrovascular diseases mainly included *Ligusticum chuanxiong* Hort, *Salvia miltiorrhiza*, *Radix paeoniae* Rubra, and *Panax notoginseng*, which could improve blood supply by inhibiting the activity of erythrocyte membrane adenosine triphosphatase (ATPase), inhibiting ATP consumption, and regulating the blood or fibrinolytic system (Li et al., 2015; Ma et al., 2020; Wu et al., 2020). According to the researched trials investigated, Xinkeshu capsule and Compound Danshen dropping pill all have as main ingredients *S. miltiorrhiza* and *P. notoginseng*. In addition, previous studies have indicated that β-blocker, angiotensin II receptor blockers and similar in WM were beneficial in protecting and improving cardiac function and inhibiting ventricular remodeling (Graudins et al., 2016). Therefore, we speculate that combined CPM and WM treatment might have better therapeutic effects and better reduce complications for greater performance in short- and long-term outcomes in previous studies (Wang et al., 2017). According to the Chinese medicine therapeutic rule “To alleviate the symptoms if the disease progresses fast and eradicate the cause if the disease develops slowly,” such a combination might have the advantage of combined therapy. In the past, it has been thought that combined CPM and WM is superior to WM alone: our review provides new evidence for this view.

Interestingly, in our study result, the combined treatment regimen of *qi*-replenishing, blood-activating, and stasis-removing and WM could significantly decrease echocardiography indices such as LVESD (SMD = −2.35; 95%CI = −3.09, −1.62) and LVMI (SMD = −1.73 95%CI = −2.92, −0.54), which were similar to previous conclusions in animal and clinical tests (Li et al., 2011; Chen et al., 2016; Chen et al., 2021). As a non-invasive technique, echocardiography could provide an effective reference and supplementary index for the clinical evaluation of cardiac function (Edyta et al., 2019). *Qi*-replenishing Chinese medicine could increase the antioxidant capacity of myocardium by regulating antioxidant-free radicals to improve heart function, thereby improving echocardiography indices (Scott et al., 2001; Xia et al., 2020). The components of *qi*-replenishing Chinese medicine for treating cardiovascular and cerebrovascular diseases mainly include *Astragalus*, Ginseng, and Dangshen, which play an important role in dilating blood vessels, breaking down cyclic adenosine, decreasing peripheral vascular resistance, inhibiting platelet aggregation, increasing the calcium inflow of cells, and activating calmodulin (Zhang and Zhang., 2017; Wang et al., 2021). In the included studies, Wenxin granule, Shexiang Tongxin dropping pill, and Yiqi Huayu capsule all contained the aforementioned *qi*-replenishing ingredients. This study, which is conducive to making clinical decisions, is the first to indicate that combined treatment regimen of CPM with the function of *qi*-replenishing, blood-activating, and stasis-removing, and WM might be optimal for improving the echocardiography indices of patients with cardiac hypertrophy. However, the original head-to-head clinical trials were few and low quality and need more related findings to support our results.

This NMA has provided new support for the hypothesis that patients with cardiac hypertrophy may obtain better clinical efficacy and related indicators from the perspective of CPM mechanism classification over other treatment regimens. However, this research also had some limitations. First, it did not directly compare treatment regimens (all studies were intervention versus WM), indicating that the strength of inference made in an NMA between different treatments was not as robust as it could be and that consistency between direct and indirect evidence could not be assessed. In addition, the number of studied samples was relatively small, resulting in potentially exaggerated therapeutic effects of treatment and preventing stronger conclusions from being made. Therefore, it will be necessary to carry out a larger sample and include more diverse Chinese medicine clinical trials to evaluate the long-term effect and further verify the function of each CPM-related mechanism and treatment regimen for patients with cardiac hypertrophy in future research.

## Conclusion

This NMA performed a generally comprehensive evaluation of CPM-related treatments for cardiac hypertrophy on different clinical outcomes. Our results indicated that the combined treatment regimen BASR-CPM and WM may exhibit outstanding efficacy compared with other treatment regimens in improving clinical efficacy for patients with cardiac hypertrophy. The QR&BASR-CPM and WM combination may be beneficial in decreasing LVESD and LVMI. Although the current estimated effects of most CPMs for cardiac hypertrophy are significant and clinically relevant, the design and included original trials are not high quality and have an unclear bias of risk. Thus, larger sample sizes and higher quality RCTs are needed to confirm and support this NMA.

## Data Availability

The original contributions presented in the study are included in the article/Supplementary Material; further inquiries can be directed to the corresponding author.

## Glossary

BASR-CPM: blood-activating and stasis-removing Chinese patent medicine
CBM: China Biology Medicine disc
CNKI: China National Knowledge Infrastructure
CPM: Chinese patent medicine
CI: confidence interval
CG: control group
CRP: C-reactive protein
HC-CPM: heat-clearing Chinese patent medicine
VIP: Information Resource Integration Service Platform
IG: intervention group
KWYR-CPM: kidney-warming and *yang*-restoring Chinese patent medicine
LVEF: left ventricular ejection fraction
LVEDD: left ventricular end-diastolic dimension
LVESD: left ventricular end-systolic dimension
LVMI: left ventricular mass index
NMA: network meta-analysis
NA: not available
non-RCTs: non-randomized controlled trials
NT-proBNP: N-terminal proBNP
OR: odds ratio
QR-CPM: *Qi*-replenishing Chinese patent medicine
QR&BASR-CPM: *Qi*-replenishing, blood-activating, and stasis-removing Chinese patent medicine
RCT: randomized controlled trial
PRISMA: Preferred Reporting Items for Systematic Reviews and Meta-Analyses
SD: standard deviation
SMD: standardized mean deviation
SUCRA: surface-under-the-cumulative-ranking curves
THSWT: Taohong Siwu decoction
TCM: traditional Chinese medicine
Wanfang Data: Wanfang Data Knowledge Service Platform
WM: Western medicine
6-MWT: Six-minute walk test
