# Supplementary material for: Comparative clinical-related outcomes of Chinese patent medicines for cardiac hypertrophy: A systematic review and network meta-analysis of randomized clinical trials
Source: Front Pharmacol. 2023 Jan 23;14:963099. doi: 10.3389/fphar.2023.963099 (PMC9900025; doi:10.3389/fphar.2023.963099)
Supplement: Supplementary file 1 [file DataSheet1.pdf]

Supplementary Material

Appendix S1 PRISMA checklist

Section and Topic	Item #	Checklist item	Location where item is reported
**TITLE**			
Title	1	Identify the report as a systematic review.	P1
**ABSTRACT**			
Abstract	2	See the PRISMA 2020 for Abstracts checklist.	P5
**INTRODUCTION**			
Rationale	3	Describe the rationale for the review in the context of existing knowledge.	P3-5
Objectives	4	Provide an explicit statement of the objective(s) or question(s) the review addresses.	P3-5
**METHODS**			
Eligibility criteria	5	Specify the inclusion and exclusion criteria for the review and how studies were grouped for the syntheses.	P5-6
Information sources	6	Specify all databases, registers, websites, organisations, reference lists and other sources searched or consulted to identify studies. Specify the date when each source was last searched or consulted.	P5
Search strategy	7	Present the full search strategies for all databases, registers and websites, including any filters and limits used.	P5
Selection process	8	Specify the methods used to decide whether a study met the inclusion criteria of the review, including how many reviewers screened each record and each report retrieved, whether they worked independently, and if applicable, details of automation tools used in the process.	P7

Section and Topic	Item #	Checklist item	Location where item is reported
Data collection process	9	Specify the methods used to collect data from reports, including how many reviewers collected data from each report, whether they worked independently, any processes for obtaining or confirming data from study investigators, and if applicable, details of automation tools used in the process.	P7
Data items	10a	List and define all outcomes for which data were sought. Specify whether all results that were compatible with each outcome domain in each study were sought (e.g. for all measures, time points, analyses), and if not, the methods used to decide which results to collect.	P6
	10b	List and define all other variables for which data were sought (e.g. participant and intervention characteristics, funding sources). Describe any assumptions made about any missing or unclear information.	P19
Study risk of bias assessment	11	Specify the methods used to assess risk of bias in the included studies, including details of the tool(s) used, how many reviewers assessed each study and whether they worked independently, and if applicable, details of automation tools used in the process.	P8
Effect measures	12	Specify for each outcome the effect measure(s) (e.g. risk ratio, mean difference) used in the synthesis or presentation of results.	P7-8
Synthesis methods	13a	Describe the processes used to decide which studies were eligible for each synthesis (e.g. tabulating the study intervention characteristics and comparing against the planned groups for each synthesis (item #5)).	P24-25
	13b	Describe any methods required to prepare the data for presentation or synthesis, such as handling of missing summary statistics, or data conversions.	P7
	13c	Describe any methods used to tabulate or visually display results of individual studies and syntheses.	P7
	13d	Describe any methods used to synthesize results and provide a rationale for the choice(s). If meta-analysis was performed, describe the model(s), method(s) to identify the presence and extent of statistical heterogeneity, and software package(s) used.	P7-8
	13e	Describe any methods used to explore possible causes of heterogeneity among study results (e.g. subgroup analysis, meta-regression).	P7-8
	13f	Describe any sensitivity analyses conducted to assess robustness of the synthesized results.	N/A

Section and Topic	Item #	Checklist item	Location where item is reported
Reporting bias assessment	14	Describe any methods used to assess risk of bias due to missing results in a synthesis (arising from reporting biases).	P8
Certainty assessment	15	Describe any methods used to assess certainty (or confidence) in the body of evidence for an outcome.	P7-8
**RESULTS**			
Study selection	16a	Describe the results of the search and selection process, from the number of records identified in the search to the number of studies included in the review, ideally using a flow diagram.	P8
	16b	Cite studies that might appear to meet the inclusion criteria, but which were excluded, and explain why they were excluded.	P8
Study characteristics	17	Cite each included study and present its characteristics.	P8-P9
Risk of bias in studies	18	Present assessments of risk of bias for each included study.	P9
Results of individual studies	19	For all outcomes, present, for each study: (a) summary statistics for each group (where appropriate) and (b) an effect estimate and its precision (e.g. confidence/credible interval), ideally using structured tables or plots.	P9
Results of syntheses	20a	For each synthesis, briefly summarise the characteristics and risk of bias among contributing studies.	P9
	20b	Present results of all statistical syntheses conducted. If meta-analysis was done, present for each the summary estimate and its precision (e.g. confidence/credible interval) and measures of statistical heterogeneity. If comparing groups, describe the direction of the effect.	P9-P15
	20c	Present results of all investigations of possible causes of heterogeneity among study results.	P10
	20d	Present results of all sensitivity analyses conducted to assess the robustness of the synthesized results.	N/A

Section and Topic	Item #	Checklist item	Location where item is reported
Reporting biases	21	Present assessments of risk of bias due to missing results (arising from reporting biases) for each synthesis assessed.	P14-P15
Certainty of evidence	22	Present assessments of certainty (or confidence) in the body of evidence for each outcome assessed.	P9-P15
**DISCUSSION**			
Discussion	23a	Provide a general interpretation of the results in the context of other evidence.	P15-P17
	23b	Discuss any limitations of the evidence included in the review.	P17
	23c	Discuss any limitations of the review processes used.	P17
	23d	Discuss implications of the results for practice, policy, and future research.	P18
**OTHER INFORMATION**			
Registration and protocol	24a	Provide registration information for the review, including register name and registration number, or state that the review was not registered.	P5
	24b	Indicate where the review protocol can be accessed, or state that a protocol was not prepared.	
	24c	Describe and explain any amendments to information provided at registration or in the protocol.	N/A
Support	25	Describe sources of financial or non-financial support for the review, and the role of the funders or sponsors in the review.	P18
Competing interests	26	Declare any competing interests of review authors.	P18-P19
Availability of data, code and other materials	27	Report which of the following are publicly available and where they can be found: template data collection forms; data extracted from included studies; data used for all analyses; analytic code; any other materials used in the review.	P19-25

Appendix S2 The detailed retrieval terms with MeSH subject words and free words

- **The following terms were used in the search:**

"Ventricular Remodeling" OR "Remodeling, Ventricula" OR "Remodelings, Ventricular" OR "Ventricular Remodelings" OR "Ventricle Remodeling" OR "Remodeling, Ventricle" OR "Remodelings, Ventricle" OR "Ventricle Remodelings" OR "Cardiac Remodeling, Ventricular" OR "Cardiac Remodelings, Ventricular" OR "Remodeling, Ventricular Cardiac" OR "Remodelings, Ventricular Cardiac" OR "Ventricular Cardiac Remodeling" OR "Ventricular Cardiac Remodelings" OR "Myocardial Remodeling, Ventricular" OR "Myocardial Remodelings, Ventricular" OR "Remodeling, Ventricular Myocardial" OR "Remodelings, Ventricular Myocardial" OR "Ventricular Myocardial Remodeling" OR "Ventricular Myocardial Remodelings" OR "Left Ventricle Remodeling" OR "Left Ventricle Remodelings" OR "Remodeling, Left Ventricle" OR "Remodelings, Left Ventricle" OR "Ventricle Remodeling, Left" OR "Ventricle Remodelings, Left" OR "Left Ventricular Remodeling" OR "Left Ventricular Remodelings" OR "Remodeling, Left Ventricular" OR "Remodelings, Left Ventricular" OR "Ventricular Remodeling, Left" OR "Ventricular Remodelings, Left" OR "Hypertrophy, Left Ventricular" OR "Left Ventricular Hypertrophy" OR "Hypertrophies, Left Ventricular" OR "Left Ventricular Hypertrophies" OR "Ventricular Hypertrophies, Left" OR "Ventricular Hypertrophy, Left" OR "Hypertrophy, Right Ventricular" OR "Ventricular Hypertrophy, Right" OR "Right Ventricular Hypertrophy" OR "Hypertrophies, Right Ventricular" OR "Right Ventricular Hypertrophies" OR "Ventricular Hypertrophies, Right" OR "Cardiomegaly" OR "Heart Enlargement" OR "Enlargement, Heart" OR "Enlarged Heart" OR "Heart, Enlarged" OR "Cardiac Hypertrophy" OR "Cardiac Hypertrophies" OR "Hypertrophies, Cardiac" OR "Hypertrophy, Cardiac" OR "Heart Hypertrophy" OR "Heart Hypertrophies" OR "Hypertrophies, Heart" OR "Hypertrophy, Heart" OR "Cardiomyopathy, Hypertrophic" OR "Cardiomyopathies, Hypertrophic" OR "Hypertrophic Cardiomyopathies" OR "Hypertrophic Cardiomyopathy" OR "Cardiomyopathy, Hypertrophic Obstructive" OR "Cardiomyopathies, Hypertrophic Obstructive" OR "Hypertrophic Obstructive Cardiomyopathies" OR "Hypertrophic Obstructive Cardiomyopathy" OR "Obstructive Cardiomyopathies, Hypertrophic" OR "Obstructive Cardiomyopathy, Hypertrophic" OR "Ventricular Remodeling" OR "Remodelings, Ventricular" OR

“Ventricular Remodelings” OR “Ventricle Remodeling” OR “Remodeling, Ventricle OR Remodelings, Ventricle” OR “Ventricle Remodelings” OR “Cardiac Remodeling, Ventricular” OR “Cardiac Remodelings, Ventricular” OR “Remodeling, Ventricular Cardiac” OR “Remodelings, Ventricular Cardiac” OR “Ventricular Cardiac Remodeling” OR “Ventricular Cardiac Remodelings” OR “Myocardial Remodeling, Ventricular” OR “Myocardial Remodelings, Ventricular” OR “Remodeling, Ventricular Myocardial” OR “Remodelings, Ventricular Myocardial” OR “Ventricular Myocardial Remodeling” OR “Ventricular Myocardial Remodelings” OR “Left Ventricle Remodeling” OR “Left Ventricle Remodelings” OR “Remodeling, Left Ventricle” OR “Remodelings, Left Ventricle” OR “Ventricle Remodeling, Left” OR “Ventricle Remodelings, Left” OR “Left Ventricular Remodeling” OR “Left Ventricular Remodelings” OR “Remodeling, Left Ventricular” OR “Remodelings, Left Ventricular” OR “Ventricular Remodeling, Left” OR “Ventricular Remodelings, Left” OR “Endocardial Fibrosis” OR “Endomyocardial Fibroses” OR “Fibroses, Endomyocardial” OR “Fibrosis, Endomyocardial” **AND** “Tablets” OR “Tablet” OR “pian” OR “san” OR “Capsules” OR “Capsule” OR “Microcapsules” OR “Microcapsule” OR “jiaonang” OR “potions” OR “chongji” OR “oral liquid” OR “koufuye” OR “pill” OR “wan” OR “Chinese proprietary herbal medicines (CPHMs)” OR “keli” **AND** “Random Allocation” OR “placebos” OR “placebos” OR “Clinical Trials, Randomized” OR “Trials, Randomized Clinical” OR “Controlled Clinical Trials, Randomized” OR “randomized OR randomised” OR “randomly” OR “trial” OR “phase” OR “RCT”.

- **Take “PubMed” as an example was shown below**

#1 ((((((((((((("Cardiomyopathy, Hypertrophic, Familial"[Mesh]) OR ((((((((((((((((((Cardiomyopathy, Familial Hypertrophic[Title/Abstract]) OR (Familial Hypertrophic Cardiomyopathies[Title/Abstract])) OR (Hypertrophic Cardiomyopathies, Familial[Title/Abstract])) OR (Hypertrophic Cardiomyopathy, Familial[Title/Abstract])) OR (Ventricular Hypertrophy, Hereditary[Title/Abstract])) OR (Asymmetric Septal Hypertrophy, Familial[Title/Abstract])) OR (Hereditary Ventricular Hypertrophy[Title/Abstract])) OR (Hereditary Ventricular Hypertrophies[Title/Abstract])) OR (Hypertrophies, Hereditary Ventricular[Title/Abstract])) OR (Hypertrophy, Hereditary Ventricular[Title/Abstract])) OR (Ventricular Hypertrophies, Hereditary[Title/Abstract])) OR (Familial

Hypertrophic Cardiomyopathy[Title/Abstract])) OR (Ventricular Hypertrophy, Familial[Title/Abstract])) OR (Familial Ventricular Hypertrophies[Title/Abstract])) OR (Familial Ventricular Hypertrophy[Title/Abstract])) OR (Hypertrophy, Familial Ventricular[Title/Abstract])) OR (Ventricular Hypertrophies, Familial[Title/Abstract])) OR (Obstructive Asymmetric Septal Hypertrophy[Title/Abstract])) OR (Hypertrophic Subaortic Stenosis, Idiopathic[Title/Abstract]))) OR ("Hypertrophy, Right Ventricular"[Mesh])) OR ("Hypertrophy, Left Ventricular"[Mesh])) OR (((((Left Ventricular Hypertrophy[Title/Abstract]) OR (Hypertrophies, Left Ventricular[Title/Abstract])) OR (Left Ventricular Hypertrophies[Title/Abstract])) OR (Ventricular Hypertrophies, Left[Title/Abstract])) OR (Ventricular Hypertrophy, Left[Title/Abstract]))) OR (((((Ventricular Hypertrophy, Right[Title/Abstract]) OR (Right Ventricular Hypertrophy[Title/Abstract])) OR (Hypertrophies, Right Ventricular[Title/Abstract])) OR (Right Ventricular Hypertrophies[Title/Abstract])) OR (Ventricular Hypertrophies, Right[Title/Abstract]))) OR ("Cardiomegaly"[Mesh])) OR ((((((((((((Heart Enlargement[Title/Abstract]) OR (Enlargement, Heart[Title/Abstract])) OR (Enlarged Heart[Title/Abstract])) OR (Heart, Enlarged[Title/Abstract])) OR (Cardiac Hypertrophy[Title/Abstract])) OR (Cardiac Hypertrophies[Title/Abstract])) OR (Hypertrophies, Cardiac[Title/Abstract])) OR (Hypertrophy, Cardiac[Title/Abstract])) OR (Heart Hypertrophy[Title/Abstract])) OR (Heart Hypertrophies[Title/Abstract])) OR (Hypertrophies, Heart[Title/Abstract])) OR (Hypertrophy, Heart[Title/Abstract]))) OR ("Cardiomyopathy, Hypertrophic"[Mesh])) OR ((((((((Cardiomyopathies, Hypertrophic[Title/Abstract]) OR (Hypertrophic Cardiomyopathies[Title/Abstract])) OR (Hypertrophic Cardiomyopathy[Title/Abstract])) OR (Cardiomyopathy, Hypertrophic Obstructive[Title/Abstract])) OR (Hypertrophic Obstructive Cardiomyopathies[Title/Abstract])) OR (Hypertrophic Obstructive Cardiomyopathy[Title/Abstract])) OR (Obstructive Cardiomyopathies, Hypertrophic[Title/Abstract])) OR (Obstructive Cardiomyopathy, Hypertrophic[Title/Abstract]))) OR ("Ventricular Remodeling"[Mesh])) OR ((((((((((((((((((((((((((Ventricle Remodeling[Title/Abstract]) OR (Ventricle Remodelings[Title/Abstract])) OR (Cardiac Remodeling, Ventricular[Title/Abstract])) OR (Cardiac Remodelings, Ventricular[Title/Abstract])) OR (Remodeling, Ventricular Cardiac[Title/Abstract])) OR (Remodelings, Ventricular Cardiac[Title/Abstract])) OR (Ventricular Cardiac Remodeling[Title/Abstract])) OR (Ventricular Cardiac

Remodelings[Title/Abstract])) OR (Myocardial Remodeling, Ventricular[Title/Abstract])) OR (Myocardial Remodelings, Ventricular[Title/Abstract])) OR (Remodeling, Ventricular Myocardial[Title/Abstract])) OR (Remodelings, Ventricular Myocardial[Title/Abstract])) OR (Ventricular Myocardial Remodeling[Title/Abstract])) OR (Ventricular Myocardial Remodelings[Title/Abstract])) OR (Left Ventricle Remodeling[Title/Abstract])) OR (Left Ventricle Remodelings[Title/Abstract])) OR (Remodeling, Left Ventricle[Title/Abstract])) OR (Remodelings, Left Ventricle[Title/Abstract])) OR (Ventricle Remodeling, Left[Title/Abstract])) OR (Ventricle Remodelings, Left[Title/Abstract])) OR (Left Ventricular Remodeling[Title/Abstract])) OR (Left Ventricular Remodelings[Title/Abstract])) OR (Remodeling, Left Ventricular[Title/Abstract])) OR (Remodelings, Left Ventricular[Title/Abstract])) OR (Ventricular Remodeling, Left[Title/Abstract])) OR (Ventricular Remodelings, Left[Title/Abstract]))) OR ("Endomyocardial Fibrosis"[Mesh])) OR (((Endomyocardial Fibroses[Title/Abstract]) OR (Fibroses, Endomyocardial[Title/Abstract])) OR (Fibrosis, Endomyocardial[Title/Abstract]))

#2 (((("Tablets"[Mesh]) OR ((Tablet[Title/Abstract]) OR (pian[Title/Abstract]))) OR ("Capsules"[Mesh])) OR ((((Capsule[Title/Abstract]) OR (Microcapsules[Title/Abstract])) OR (Microcapsule[Title/Abstract])) OR (jiaonang[Title/Abstract]))) OR ((((((((san[Title/Abstract]) OR (potions[Title/Abstract])) OR (chongji[Title/Abstract])) OR (oral liquid[Title/Abstract])) OR (koufuye[Title/Abstract])) OR (pill[Title/Abstract])) OR (wan[Title/Abstract])) OR (keli[Title/Abstract])) OR (Chinese proprietary herbal medicines[Title/Abstract]))

#3 (((((((((((Random Allocation[Title/Abstract]) OR (placebos[Title/Abstract])) OR (Clinical Trials, Randomized[Title/Abstract])) OR (Trials, Randomized Clinical[Title/Abstract])) OR (Controlled Clinical Trials, Randomized[Title/Abstract])) OR (Allocation, Random[Title/Abstract])) OR (Randomization[Title/Abstract])) OR (randomized[Title/Abstract])) OR (randomised[Title/Abstract])) OR (randomly[Title/Abstract])) OR (trial[Title/Abstract])) OR (phase[Title/Abstract])) OR (rct[Title/Abstract])

#4 #1 and #2 and #3

A

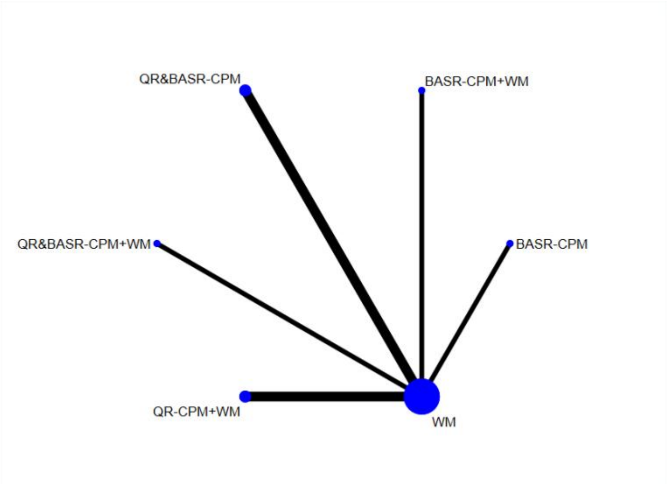

B

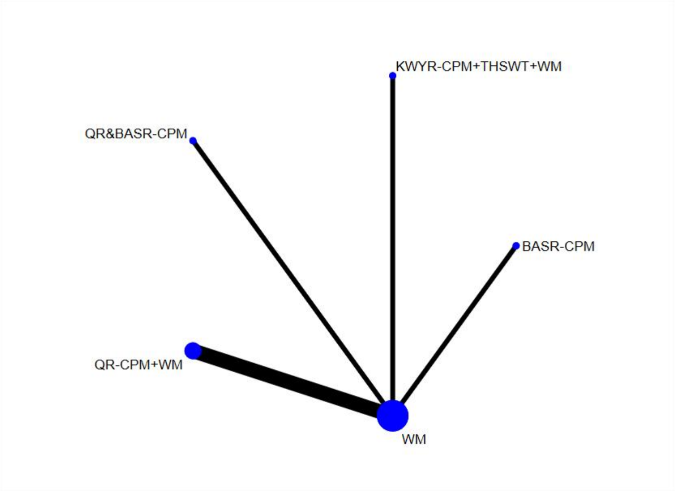

Figure 1. The network map of secondary outcomes

Notes: **(A):** NT-proBNP; **(B):** 6-MWT

1: WM; **2:** QR-CPM; **3:** QR-CPM+WM; **4:**BASR-CPM; **5:** BASR-CPM+WM; **6:** QR&BASR-CPM; **7:** QR&BASR-CPM+WM; **8:** KWYR-CPM+THSWT+WM; **9:** HC-CPM+WM

A. The forest plot of Clinical Efficacy

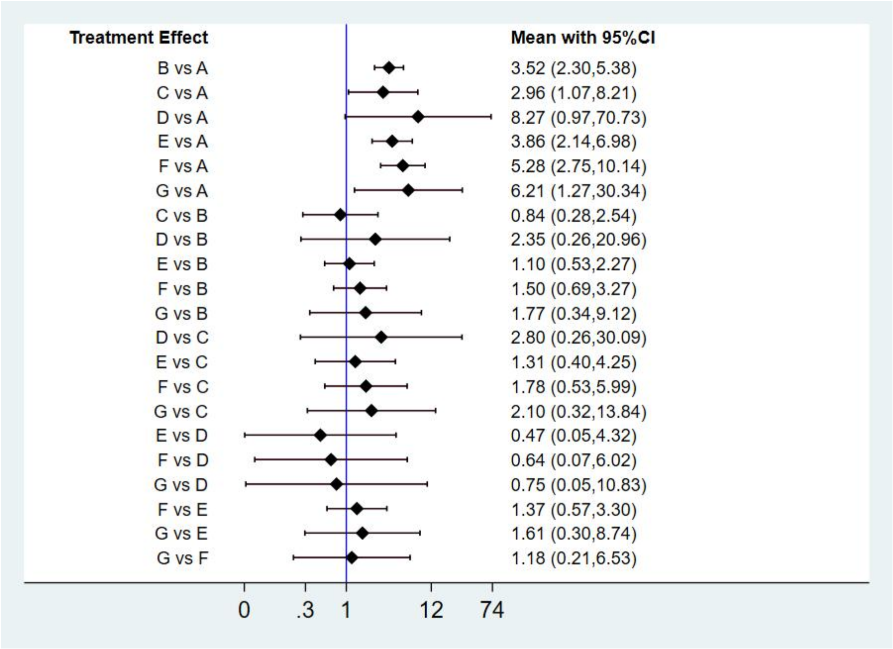

Note: A: WM; **B:** QR-CPM+WM; **C:** BASR-CPM; **D:** BASR-CPM+WM; **E:** QR&BASR-CPM; **F:** QR&BASR-CPM+WM; **G:** HC-CPM+WM.

B. The forest plot of LVEF

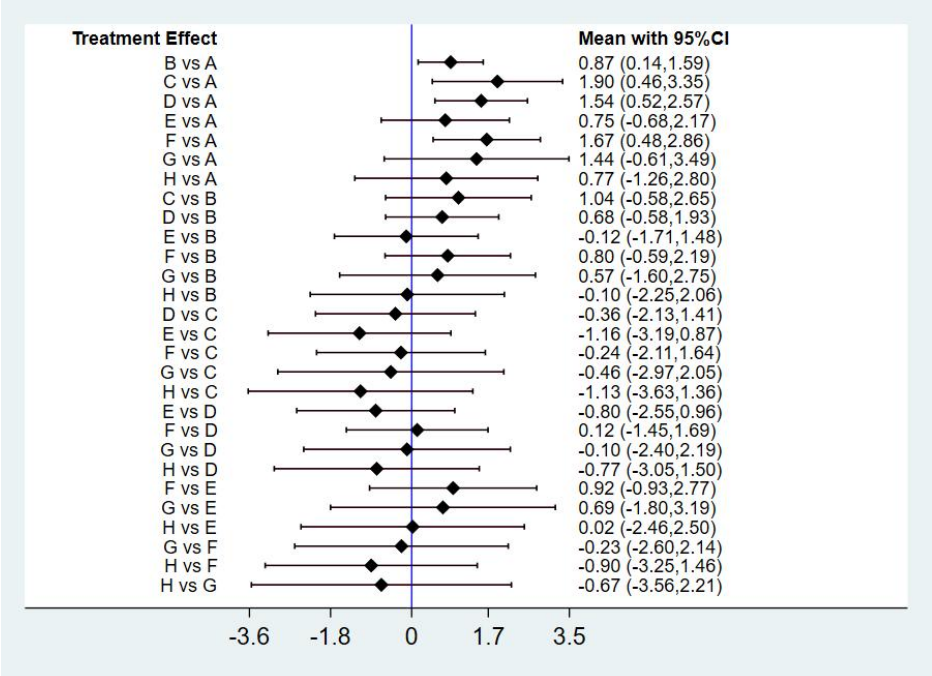

Note: A: WM; **B:** QR-CPM+WM; **C:**BASR-CPM; **D:** BASR-CPM+WM; **E:** QR&BASR-CPM; **F:** QR&BASR-CPM+WM; **G:** KWYR-CPM+THSWT+WM; **H:** HC-CPM+WM

C. The forest plot of LVEDD

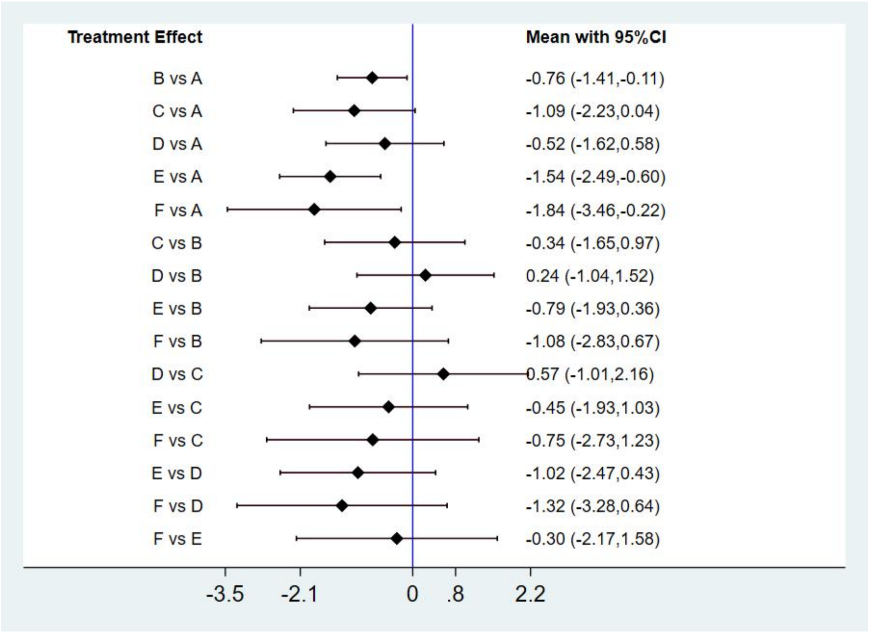

Note: A: WM; **B:** QR-CPM+WM; **C:** BASR-CPM+WM; **D:** QR&BASR-CPM; **E:** QR&BASR-CPM+WM; **F:** KWYR-CPM+THSWT+WM;

D. The forest plot of LVESD

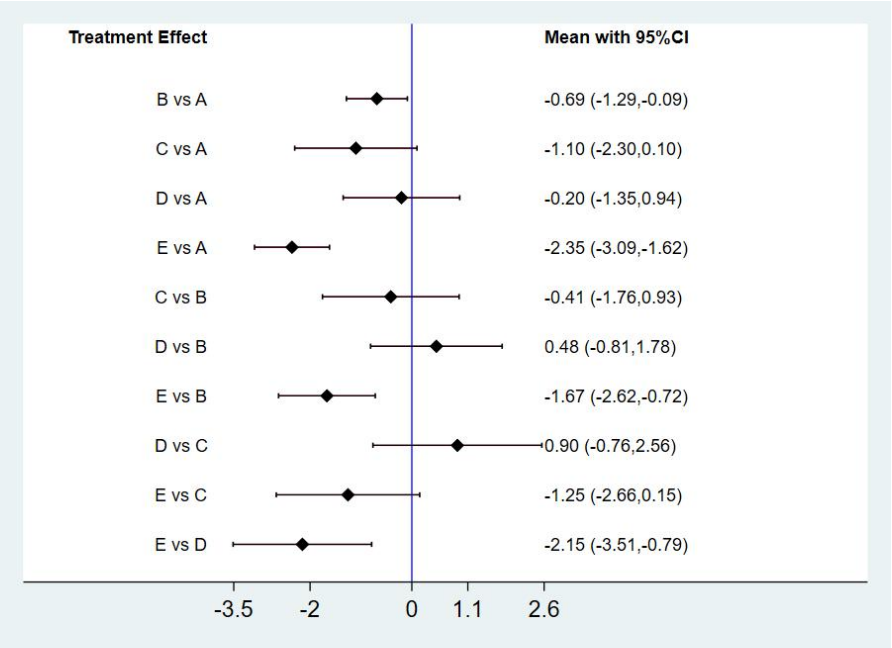

Note: A: WM; **B:** QR-CPM+WM; **C:** BASR-CPM+WM; **D:** QR&BASR-CPM; **E:** QR&BASR-CPM+WM;

E. The forest plot of LVMI

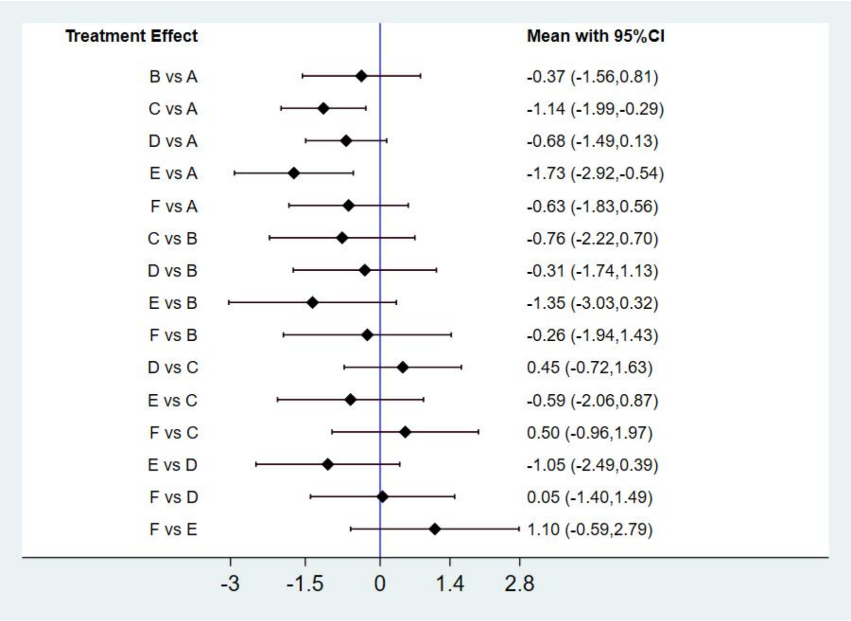

Note: **A:** WM; **B:** QR-CPM+WM; **C:** BASR-CPM+WM; **D:** QR&BASR-CPM; **E:** QR&BASR-CPM+WM; **F:** KWYR-CPM+THSWT+WM;

F. The forest plot of CRP

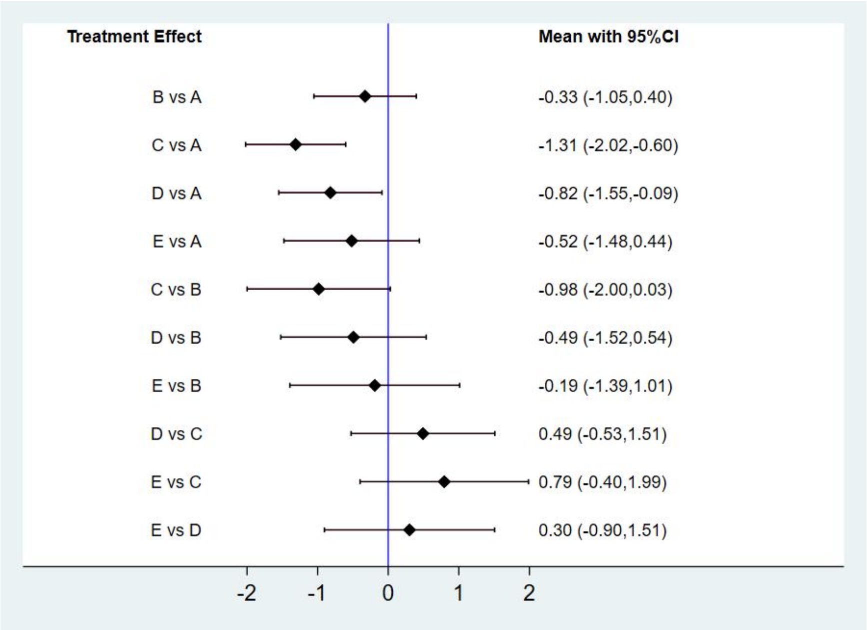

Note: A: WM; **B:** QR-CPM+WM; **C:**BASR-CPM; **D:** BASR-CPM+WM; **E:** QR&BASR-CPM;

G. The forest plot of NT-proBNP

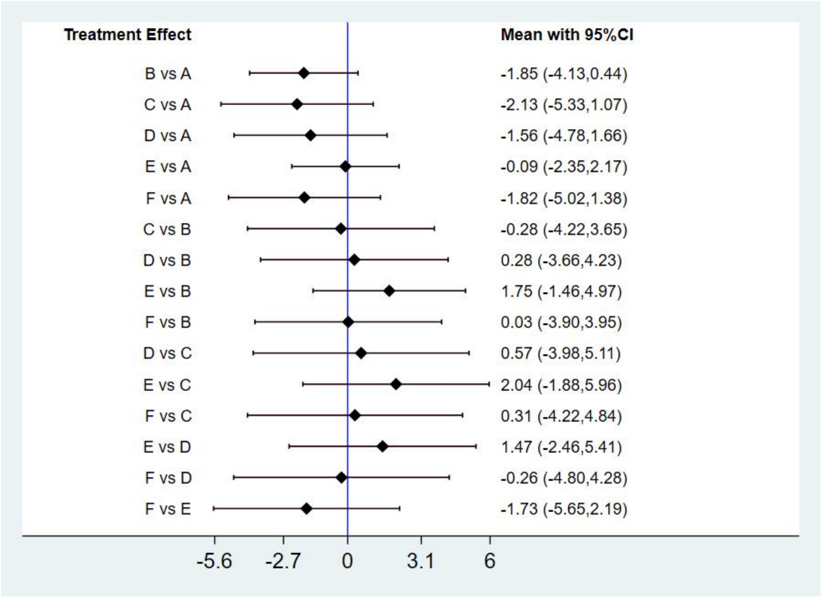

Note: A: WM; **B:** QR-CPM+WM; **C:**BASR-CPM; **D:** BASR-CPM+WM; **E:** QR&BASR-CPM; **F:** QR&BASR-CPM+WM;

H. The forest plot of 6-MWT

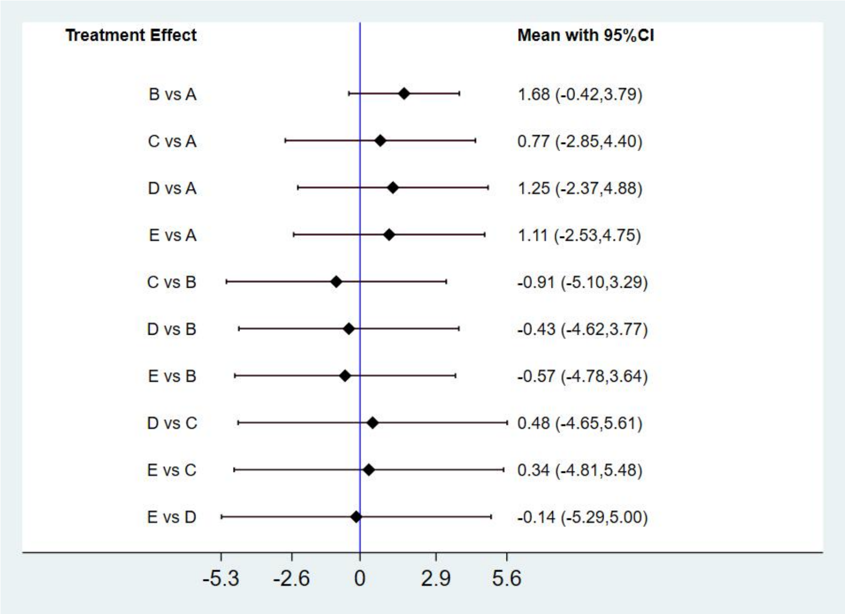

Note: A: WM; B: QR-CPM+WM; C:BASR-CPM; D: QR&BASR-CPM; E: KWYR-CPM+THSWT+WM;

Figure 2. The forest plot between treatment regimen for each outcome

A. The SUCRE of Clinical Efficacy

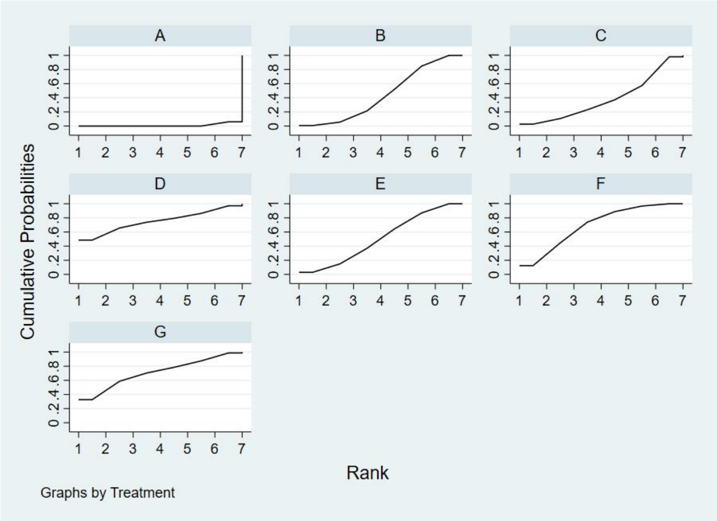

B. The SUCRE of LVEF

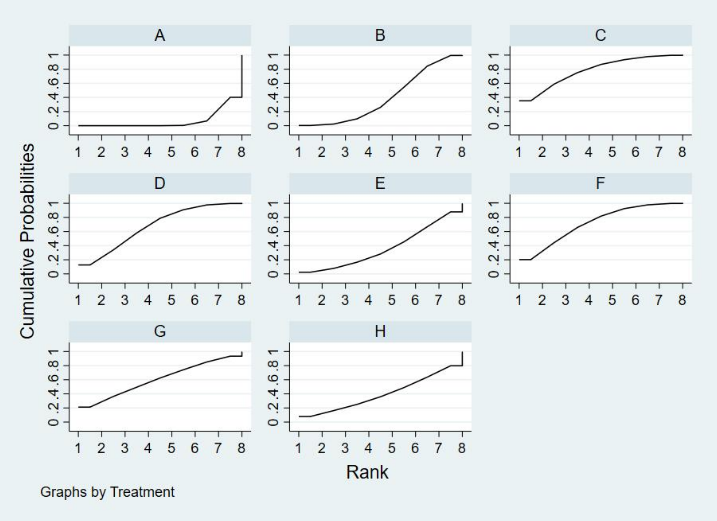

C. The SUCRE of LVEDD

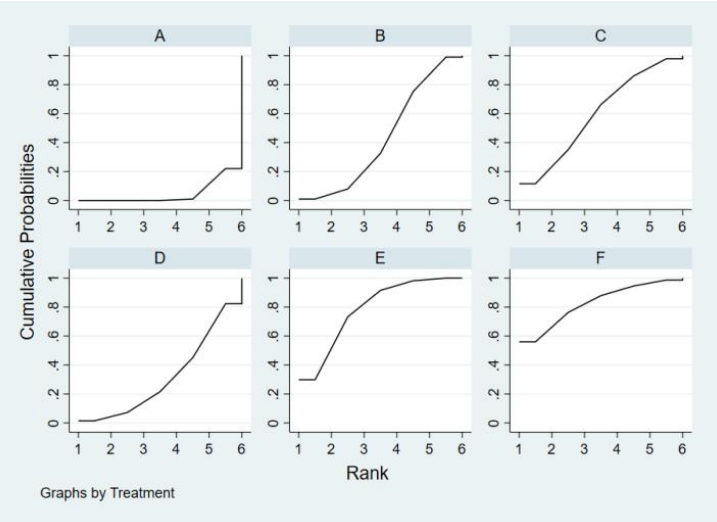

D.The SUCRE of LVESD

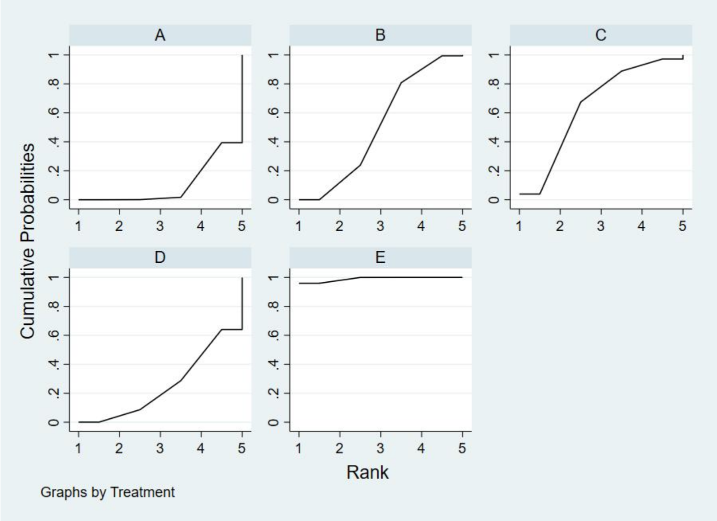

E. The SUCRE of LVMI

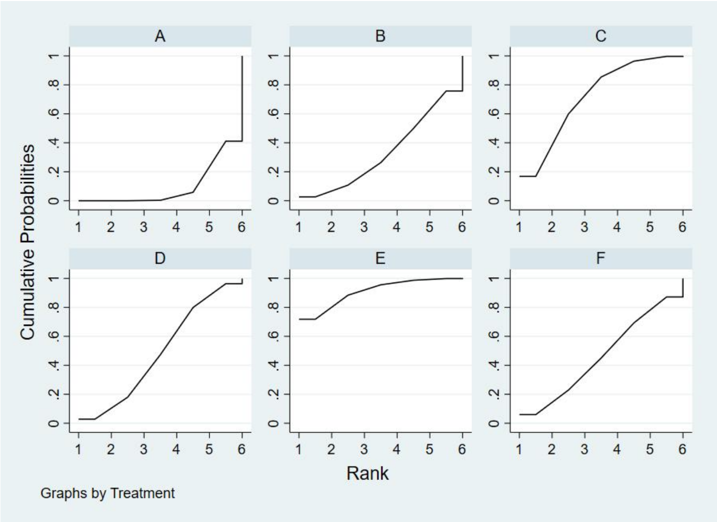

F. The SUCRE of CRP

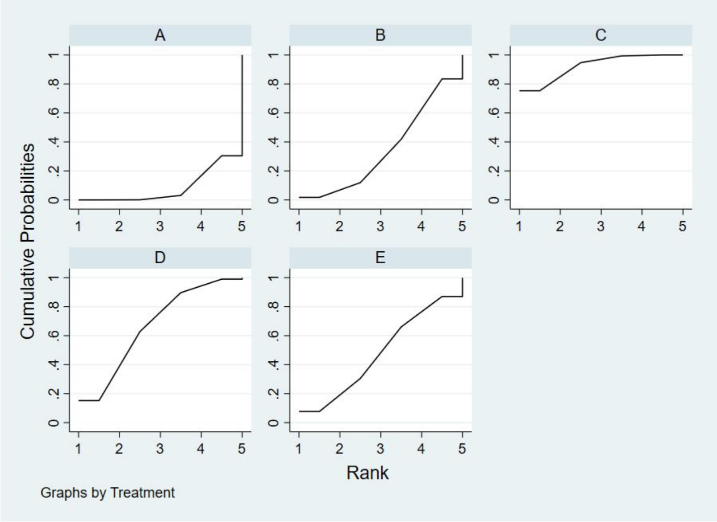

G. The SUCRE of NT-proBNP

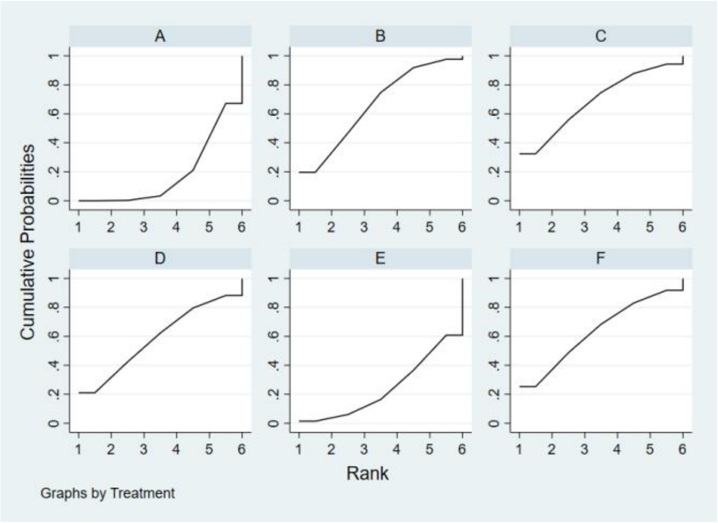

H. The SUCRE of 6-MWT

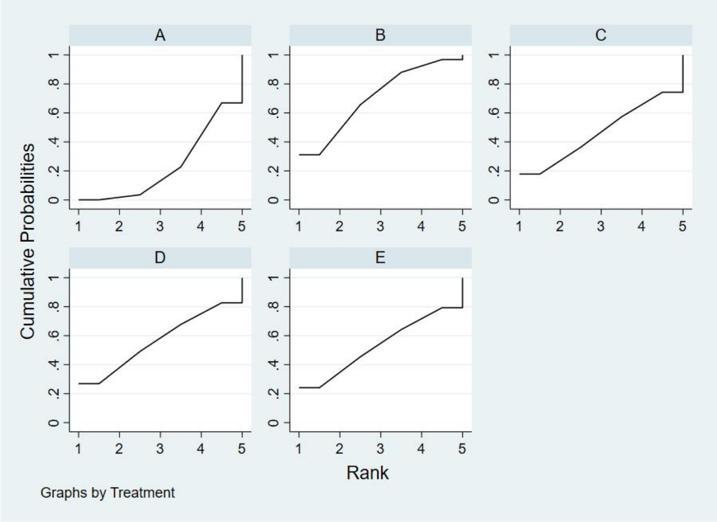

Figure 3. The surface under the cumulative ranking for each outcomes

Note: The specific treatment regimens information are commented in the same way as in Figure 2.

A. The Funnel Plots of LVEF

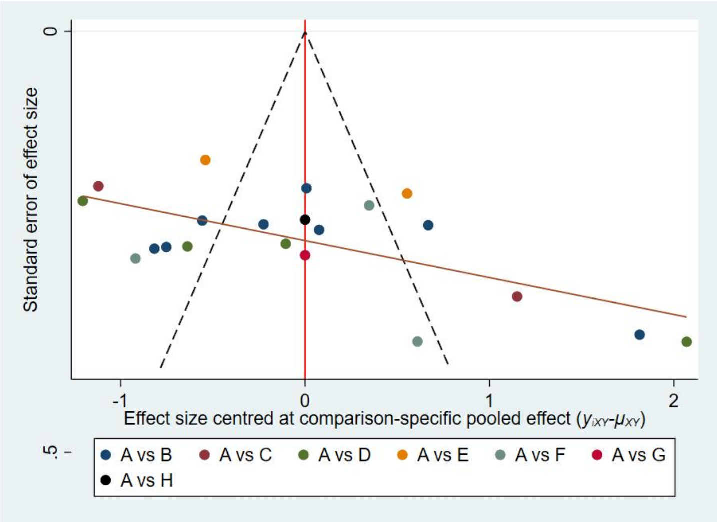

B. The Funnel Plots of LVEDD

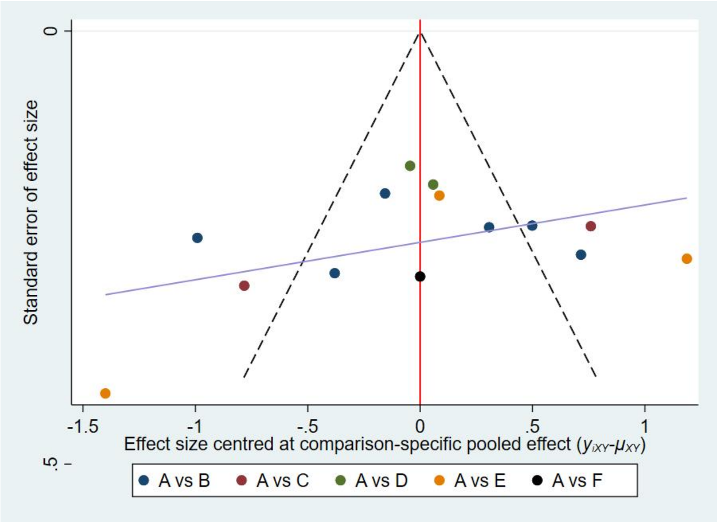

Figure 4. The funnel plots of LVEF and LVEDD

Note: The specific treatment regimens information are commented in the same way as in Figure 2B and C.
